# Japan considered from the hypothesis of farmer/language spread

**DOI:** 10.1017/ehs.2020.7

**Published:** 2020-05-05

**Authors:** Elisabeth de Boer, Melinda A. Yang, Aileen Kawagoe, Gina L. Barnes

**Affiliations:** 1 Faculty of East Asian Studies, Ruhr-Universität Bochum, Bochum, Germany; 2 Department of Biology, University of Richmond, Richmond, Virginia, USA; 3 Department of Social Studies, New International School of Japan, Tokyo, Japan; 4 SOAS, University of London, London, UK

**Keywords:** Japanese language, Yayoi agriculture, Jōmon genetics, language/farming hypothesis, Mumun–Yayoi migrations

## Abstract

Formally, the Farming/Language Dispersal hypothesis as applied to Japan relates to the introduction of agriculture and spread of the Japanese language (between ca. 500 BC–AD 800). We review current data from genetics, archaeology, and linguistics in relation to this hypothesis. However, evidence bases for these disciplines are drawn from different periods. Genetic data have primarily been sampled from present-day Japanese and prehistoric Jōmon peoples (14,000–300 BC), preceding the introduction of rice agriculture. The best archaeological evidence for agriculture comes from western Japan during the Yayoi period (ca. 900 BC–AD 250), but little is known about northeastern Japan, which is a focal point here. And despite considerable hypothesizing about *prehistoric* language, the spread of *historic* languages/ dialects through the islands is more accessible but difficult to relate to prehistory. Though the lack of Yayoi skeletal material available for DNA analysis greatly inhibits direct study of how the pre-agricultural Jōmon peoples interacted with rice agriculturalists, our review of Jōmon genetics sets the stage for further research into their relationships. Modern linguistic research plays an unexpected role in bringing Izumo (Shimane Prefecture) and the Japan Sea coast into consideration in the populating of northeastern Honshu by agriculturalists beyond the Kantō region.

## Graphical abstract

**Figure:**
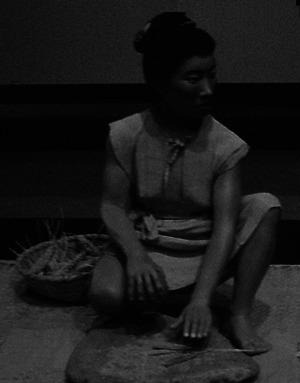

**Media summary:** How Jōmon genetics, Mumun migrations, and Japanese dialect differences inform farming/language spread hypothesis in Japan

## Introduction

**Figure 1.**
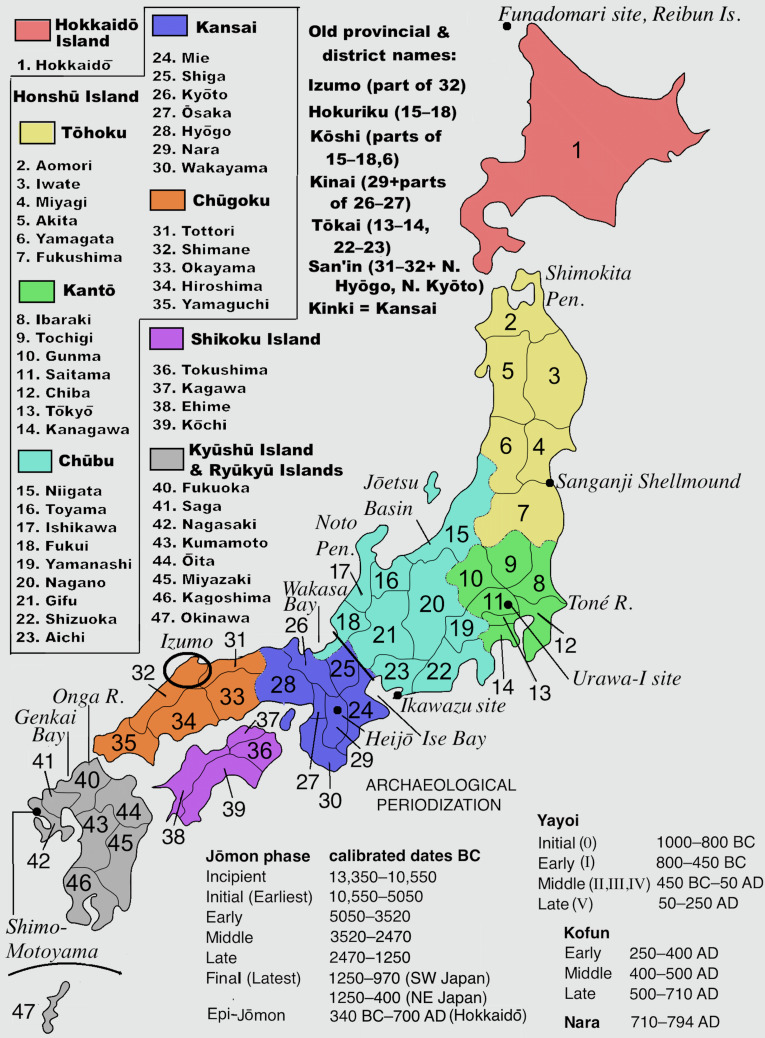
Map of Japan: prefectures, districts, and placenames mentioned in the text, with Japanese archaeological periodizations. Sources: Periodization from Barnes (2015), tables 1.6, 1.8. Map by TheOtherJesse [public domain], via Wikimedia Commons, modified by GLB [https://commons.wikimedia.org/wiki/File:Regions_and_Prefectures_of_Japan.svg]

The Jōmon period (see Figure 1) is one of the longest post-Palaeolithic archaeological periods in the world, sustained by a hunting–gathering–fishing–horticultural subsistence pattern (Habu, 2004). The introduction of rice agriculture into the Genkai Bay area of North Kyushu (Figure 1) from around 1000 BC marks the beginning of the Yayoi period. Wet-rice technology was introduced by Mumun (‘plain pottery’) culture migrants from the southern Korean Peninsula relatively early, ca. 1000 BC, in the Peninsula's Mumun period (1500–300 BC). These migrants were *not* Yayoi, they were Mumun.

The term Yayoi has four uses, which can create much confusion. First, it is the designation of the period beginning with the introduction of rice agriculture around 1000 BC until the advent of the Mounded Tomb Culture in the third century AD. Yayoi is a period designation exclusive to Japan; it includes both farmers and hunter–gatherers and entails the agricultural transition in a time-transgressive and regionally disparate process. Second, ‘Yayoi people’ may refer to anyone living in the Japanese Islands in the Yayoi period, or third, Yayoi may refer specifically to admixed people (Mumun + Jōmon in varying in proportions and across great distances). Fourth, Yayoi may indicate acculturation: the adoption of (rice) agriculture (and other continental material culture) by Jōmon-lineage people in the Yayoi period. All of these conflicting aspects of Yayoi must be kept in mind and clearly defined in any discussion.

Upon the Mumun migrants’ arrival, it is assumed from artefactual, skeletal morphological, and genetic evidence that many took partners from the resident Jōmon population, leading to mixed descendants – and to cultural transformation. These generational ‘admixed’ farmers then spread out from North Kyushu into the rest of the Japanese Islands, meeting more local Jōmon-lineage peoples with further admixing taking place. It is common in the literature to label the admixed farmers as ‘Yayoi’ because they carried the new culture based on rice, while increasingly recognizing that Jōmon-lineage peoples (distinguished as Epi-Jōmon in the north, but included within ‘Yayoi’ in the south) survived well into historical times without substantial admixture. The mixing of these two populations is recognized as the Dual Structure Hypothesis of the modern mainland Japanese population (Hanihara, 1991, 2000).

The first section below reports on Jōmon genetics as a basis for further research on Yayoi admixtures. Despite the 13,000-year Jōmon history entailing multiple cultural formations and temporal trends, archaeologists tend to treat the Jōmon as a homogeneous people unadmixed with mainland East Asians and Austronesians since their separation from each other in the Palaeolithic. Genetic research is disabusing us of that approach, revealing potential Jōmon contacts with coastal mainland East Asians before the arrival of Mumun migrants; these data mirror archaeological evidence of contact. This is enlightening for its own sake, particularly in tracing connections between the Jōmon and continental peoples. However, in terms of investigating Yayoi admixtures, it makes the issue much more complicated. As more Yayoi-period genomic data becomes available, we may then be able to track the regional spread of continental genes through the Japanese archipelago. The addition of new Yayoi-period genomic data is vital, as new waves of immigration from the Korean Peninsula in the succeeding Kofun and Nara periods have enhanced the continental contribution in the modern Japanese, making it difficult to use present-day genomes to effectively study population movement and admixture in the Yayoi period.

The second section focusses on the spread of agriculture from west to east through the Japanese Islands. The major topic of study in this spread has been the adoption of irrigated wet-rice agriculture. It has been assumed that this technology was spread by admixed farmer-migrants. In contrast, new studies are documenting instances of non-irrigated (swamp, dryland) rice and millet cultivation that may be earlier (e.g. Nasu & Momohara, 2016; Shitara & Takashe [sic], 2014). These authors assume that local Final Jōmon and Epi-Jōmon peoples were the cultivators, although some may have become farmers of irrigated wet rice later. The relationships between cultivation technologies and ethnic populations are often unclear, making the next step in the analysis, on language spread, difficult.

The third section deals with language spread. Prehistoric language as such is inaccessible, but the fact that nowadays all languages on the islands except Ainu are related to Japanese makes it absolutely clear that the population that brought wet-rice agriculture to Japan must have been speakers of proto-Japanese. (Proto-Japanese is the ancestral language from which all modern varieties of Japanese descended.) Proto-Japanese split into several dialects, and then Ryūkyūan split off from the Kyūshū dialect. Evidence for the latter scenario has been growing in recent years. (For an overview see De Boer 2017.) Although the Ryūkyūan varieties form independent languages now, their split from the Kyūshū dialect makes them in origin dialects of Japanese.

Proto-Japanese is postulated to have developed on the continent (Whitman, 2011; Miyamoto, 2016; Robbeets, 2005, 2017), and it is hypothesized to have had spread throughout the archipelago with the admixed Yayoi people practising irrigated rice agriculture. This accords well with the farming/language dispersal hypothesis (Renfrew, 1987; Bellwood & Renfrew, 2002). Problems, however, include exactly when and how this spread took place, whether it was indeed only associated with rice agriculture or also with other grain crops, and what Jōmon or Epi-Jōmon language(s) might have been replaced or pushed out by which dialect of Japanese. With the general lack of Yayoi DNA and written linguistic data, it is difficult to match food production techniques, genomic identity and language type.

## Jōmon genetics

The transition from the Jōmon to the Yayoi periods marked a transition in the material culture of Japan, with the appearance and increase of tools, artefacts and archaeobotanical remains associated with agriculture. Studies have long associated this transition with the migration of Mumun people through the Korean peninsula into the closest island in the Japanese archipelago, Kyushu (Hammer & Horai, 1995; Hanihara, 1991). Early assessments using cranial and dental morphology and genetics of humans and their commensals led to the Dual Structure model (Hanihara, 1991), for which a key component is the argument that present-day Japanese are a mixture of populations indigenous to Japan since the Palaeolithic and later migrants from mainland Asia. Many studies, mostly using present-day genomes of indigenous populations in the archipelago (i.e. Ainu, Ryūkyū) as a proxy for Jōmon-related ancestry, found genetic patterns that suggest that the present-day Japanese populations are admixed, supporting that aspect of the Dual Structure model (Jeong, Nakagome, & Di Rienzo, 2016a; Jinam et al., 2015; Nakagome et al., 2015).

Advances in DNA sequencing methods have allowed access to genetic material for studying population demographic history in the Japanese archipelago. Uniparental markers were some of the earliest used to infer population history; these include mitochondrial DNA (mtDNA), which exists outside the nucleus, and the non-recombining portion of the Y-chromosome. The primary units considered when studying these markers are haplogroups, which are variants of a DNA segment that derives from the same common ancestor, and they can provide information on a population's genetic history.

Since uniparental transfer results in no recombination, it is straightforward to reconstruct phylogenetic trees and assign a haplogroup to the mtDNA or Y-chromosome region (Haber, Mezzavilla, Xue, & Tyler-Smith, 2016). Sets of haplogroups are now well annotated and easily identifiable in newly sequenced individuals using many tools and databases (e.g. ISOGG, 2019; Weissensteiner et al., 2016). If they are identified to be at high frequency in a localized area but in low frequency elsewhere, the origin of the haplogroup, or the common ancestor, can be inferred to have derived from that region. While the lack of recombination allows simple haplogroup assignation, it also means that it is a single data point representing population history, and stochastic events or sex-biased migration patterns can lead to inaccurate inference of the true population history (Nordborg, 1998; Rosenberg & Nordborg, 2002).

In contrast, the nuclear genome undergoes recombination, which means that small segments of each chromosome have effectively independent histories. Study of a single diploid genome (i.e. the 23 pairs of chromosomes in the cell nucleus) can be interpreted as study of small portions of thousands of different individuals, resulting in high statistical power for inferring the history of the population(s) represented by those individuals. Thus, sampling genome-wide is imperative for assessing the accuracy of the demographic relationships inferred from uniparental markers.

The ability to extract and sequence ancient DNA (aDNA) has revolutionized the study of human prehistory, allowing direct sampling of past populations and revealing genetic variation no longer observed today. Post-mortem degradation of DNA makes it very difficult to sequence aDNA, so the greater numbers of mtDNA makes it much easier to extract and sequence. The earliest ancient DNA studies in humans were for mtDNA (e.g. Krings et al., 1997), including from a Jōmon individual as early as 1989 (Horai et al., 1989) and Yayoi individuals as early as 1995 (Oota, Saitou, Matsushita, & Ueda, 1995). In fact, the majority of ancient DNA studies on Jōmon individuals have been on mtDNA (Schmidt & Seguchi, 2014).

Only recently have ancient nuclear genomes been retrieved from individuals from Japan. Ancient genomes have been published from human remains at different sites (Sanganji, Ikawazu, and Funadomari associated with Jōmon material culture; Shimo-Motoyama associated with Yayoi material culture; Figure 1). Two partial genomes sequenced from the Sanganji shellmound in Tōhoku were of 3000-year-old individuals (Kanzawa-Kiriyama et al., 2017). Genome-wide data have also been retrieved for a 2700-year-old individual from Ikawazu (McColl et al., 2018) and two 3900-year-old individuals from the Funadomori site in Hokkaido (Kanzawa-Kiriyama et al., 2019). Sampling of nuclear genomes is still sparse, but a rough understanding of the ancestry associated with the Jōmon is taking shape with each study.

Characterizing Jōmon-period ancestry is fundamental to understanding the genetic composition of humans that lived during the Yayoi period, who ostensibly represent various degrees of mixture between Jōmon and continental peoples. This in turn will improve our understanding of population migration on the archipelago and its role in the spread of agriculture across the archipelago. Here, we highlight some key features and emerging questions observed to date on Jōmon-related ancestry, which allow us to better understand the challenges (and rewards!) in interpreting the genetics of the Yayoi in future studies.

Analyses of both uniparental (Y and mtDNA) and nuclear DNA of present-day populations have highlighted that ancestry persists in Japan that is deeply diverged from ancestry found in mainland East Asian populations. The distribution of haplogroup frequencies across multiple populations is often used to determine where haplogroups originated. For example, those that occur in high frequency in present-day populations from Japan but low frequency elsewhere (e.g. N9b, M7a, G1b) were proposed as candidate haplogroups of Jōmon origin (Adachi, Shinoda, Umetsu, & Matsumura, 2009; Adachi et al., 2011; Kanzawa-Kiriyama, Saso, Suwa, & Saitou, 2013; Kivisild et al., 2002).

The most prominent of these is N9b, which is the most frequent mtDNA haplogroup assigned to Jōmon individuals. An estimate of the time to the most recent common ancestor (*t*_MRCA_) is ca. 22,000 years ago, which is older than the *t*_MRCA_ for several other haplogroups found in East Asians (Adachi et al., 2011). The non-recombining region of the Y-chromosome has also been extensively studied in Japan. Y-haplogroup D1b is exceptionally interesting as it is found in high frequency in the Japanese archipelago and the Tibetan Plateau but not elsewhere (Hammer & Horai, 1995; Tajima et al., 2004; Watanabe et al., 2019). The estimated *t*_MRCA_ for D1b is ca. 19,400 years ago (Hammer et al., 2006), similar to that estimated for N9b mtDNA. These dates, if corresponding to the expansion of Jōmon ancestry on the archipelago, suggest that Jōmon ancestry separated fairly early from mainland East Asian ancestry.

One mtDNA haplogroup found in many Jōmon-associated individuals (e.g. Kanzawa-Kiriyama et al., 2013; Adachi et al., 2011; Kim et al., 2010) as well as Yayoi-associated individuals (Igawa et al., 2009) is D4, which is widespread in northern East Asia both in past and present-day populations (e.g. Tanaka et al., 2004; Zhao et al., 2010; Hong et al., 2014; Jeong et al., 2018; Lan et al., 2019). Its widespread presence, however, suggests that it was found in the common ancestral population of all East Asians, making it difficult to evaluate its role in more recent migrations and interactions.

In genomic studies of ancient Asians, both a 40,000-year-old individual found in Tianyuan Cave, Beijing, China (Fu et al., 2013; Yang et al., 2017) and 8000-year-old individuals from Southeast Asia associated with Hòabìnhian foragers (McColl et al., 2018) show an early split time from other East Asians, including from ancient Jōmon genomes (Kanzawa-Kiriyama et al., 2017, 2019). Thus, we denote them as ‘basal Asians’. In contrast, humans living in Southeast Asia, the eastern steppes of Siberia and the southwest edges of the Tibetan Plateau from the Neolithic to historic periods (e.g. Sikora et al., 2019; Jeong et al., 2016b) share a closer relationship to present-day East Asians than the Jōmon individuals, confirming that Jōmon are distantly related to mainland East Asian ancestry (Yang, MA and Fu, Q unpublished observations).

In fact, the Jōmon sampled thus far probably separated earlier than the split time of East Asians and the early Asian population that contributed to Native Americans (Gakuhari et al., 2019; Kanzawa-Kiriyama et al., 2017, 2019). Estimates of when ancestral Native Americans separated from the East Asian lineage is ca. 36,000–25,000 years ago (Moreno-Mayar et al., 2018), and a direct estimate of the split time between the Jōmon and mainland East Asians ranges from 38,000–18,000 years ago (Kanzawa-Kiriyama et al., 2019). These estimates are broad, indicating there is still high uncertainty as to the exact date. However, studies of both uniparental and nuclear markers all highlight Jōmon ancestry separated from mainland East Asian ancestry in the Palaeolithic, with dates ranging from 38,000–18,000 years ago.

Box: Three-way Analysis of Genetic Relationships

**Figure 2.**
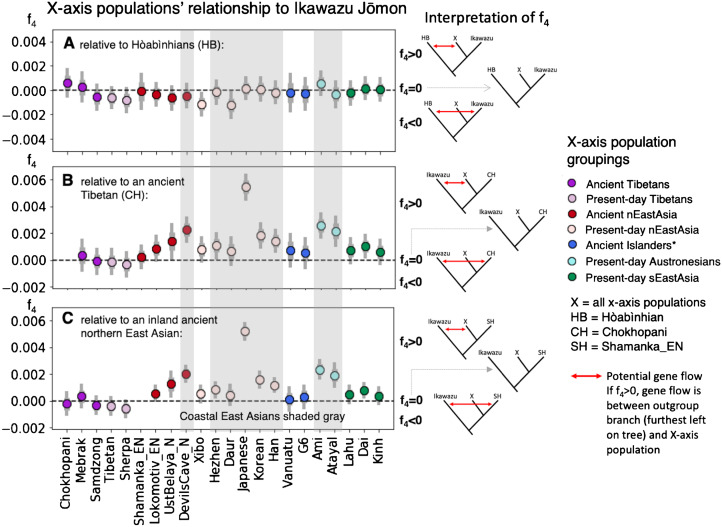
F4-tests surveying the relationship of populations with East Asian ancestry (on the *x*-axis, ‘X’) to the Ikawazu Jōmon, relative to ancient Southeast Asian foragers (Hòabìnhian, HB), an ancient Tibetan (Chokhopani, CH), and an inland ancient northern East Asian (Shamanka_EN, SH) (graphs by Melinda Yang). Numerical values for the data visualized here can be found in Table S3. F4-statistics are tests of symmetry of the form f4(Out, P1; P2, P3), where it is assumed that P2 and P3 form a clade with espect to P1. Our statistics take the form (A) f4(Mbuti, HB; Ikawazu Jōmon, ‘X’ East Asians) or (B, C) f4(Mbuti, Ikawazu Jōmon; CH or SH, ‘X’ East Asians), with the underlying tree structure verified by other genetic analyses. Central African Mbuti were used as the outgroup (Out).

*Key*: tree models showing relationships of f4 values are placed to the right of the plot: f4 > 0 corresponds to the highest tree; f4 = 0 corresponds to the middle tree (clarified by dashed grey arrow); and f4 < 0 corresponds to the lowest tree for each figure. The presence of gene flow in the model is indicated by the red arrow. A significantly positive value highlights gene flow between the outgroup (P1) and ‘X’ East Asians (P3). A significantly negative value highlights gene flow between the outgroup (P1) and the other population in the tree (P2). Two standard errors are indicated by the thin grey bar, while one standard error is indicated by the thick grey bar. Points are coloured (colour online) by region of origin, with darker colours for ancient populations and lighter colours for present-day populations. East Asians for whom all or part of their population live near coastal East Asia are shaded grey. *Ancient islanders were sampled from Southeast Asian and Southwest Pacific islands, but were previously shown to share a close relationship to present-day Austronesians (McColl et al., 2018; Skoglund et al., 2016).

*Interpretation*: in Figure 2A, 8000-year-old Hòabìnhian (HB) foragers from Laos and Malaysia are an outgroup to the Ikawazu Jōmon individual and present-day and ancient populations of East Asian ancestry. A connection between the Ikawazu Jōmon individual and the Hòabìnhians would result in a significantly negative statistic (f_4_ < 0), which is not observed. In all cases, the Ikawazu Jōmon shows no connection to Hòabìnhians (f_4_ ≈ 0).

In Figure 2B, we compare against a 3100-year-old Tibetan (Chokhopani, CH), and in Figure 2C, we compare against a 7500-year-old northern East Asian from the eastern steppe region of Siberia (Shamanka_EN, SH). The Ikawazu Jōmon is an outgroup to all East Asians, including these ancient inland East Asians. Thus, a significantly positive statistic (f_4_ > 0) indicates that the Ikawazu Jōmon shares connections with that East Asian (X) that the ancient inland East Asian Chokhopani or Shamanka_EN do not possess. No connections are observed between the Ikawazu Jōmon and ancient inland East Asians, which would be indicated by a significantly negative statistic (f_4_ < 0). A symmetric relationship to Ikawazu is observed when comparing with other non-coastal ancient and present-day East Asians (f_4_ ≈ 0). Coastal East Asians from northern and southern East Asia tend to show significantly positive results (f_4_ > 0), indicating a connection to the Ikawazu Jōmon that ancient inland East Asians do not share.

A key aspect of the observed genome-wide patterns above is that these Jōmon individuals do not share any relationship with sampled ‘basal Asians’. In admixture tests (Patterson et al., 2012), two populations that form a clade (e.g. the Jōmon and mainland present-day East Asians) may sometimes share excess ancestry with an outgroup population that potentially indicates partial shared demographic history, probably via a population closely related to the outgroup. However, Jōmon individuals show no excess connection to ‘basal Asians’, including the Southeast Asian Hòabìnhians relative to present-day East Asians, as indicated by the lack of significantly negative values in the Box (Figure 2A).

In particular, comparison of the Southeast Asian Hòabinhians from Laos and Malaysia with mainland East Asians from East Asia and a Jōmon individual (Ikawazu) shows that the East Asians and Jōmon ancestry are equidistant from Hòabìnhian ancestry (Figure 2A), indicating that the Jōmon do not share a special relationship with Hòabìnhians as previously suggested (McColl et al., 2018). Tests of genetic similarity do not show Hòabìnhians or the Jōmon sharing exceptionally high genetic similarity with each other (Figure S1). Shared ancestry between present-day Japanese and recent Southeast Asians are best explained by a common process – gene flow from mainland East Asia, a phenomenon well-characterized by tests of the Dual Structure model in Japan and by observations of gene flow into Southeast Asia (Kanzawa-Kiriyama et al., 2017, 2019; Lipson et al., 2018). In the future, individuals from eastern Eurasia will almost certainly help to clarify how Jōmon ancestry first entered the Japanese archipelago, but the current sampling has not yet shed light on who they might have been.

Connections do exist between the Jōmon and East Asians who live along the coast of East Asia. The first Jōmon mtDNA sequenced in 1989, from the Early Jōmon Urawa-I site in the Kantō region (Figure 1), shared a close relationship to present-day Southeast Asians that suggests common ancestry with Southeast Asians (Horai et al., 1989). This haplogroup, called E1a1a, is prevalent in Austronesian-speaking populations of Taiwan and Southeast Asia who share a closer relationship to East Asians than other Southeast Asians. A ca. 8000-year-old individual sampled from Liang Island off the southeast coast of China in Fujian province also shares this haplogroup (Ko et al., 2014). Study of the Liang Island individual's nuclear genome indicates a close genetic relationship to Austronesians (Yang, MA and Fu, Q unpublished observations). Present-day Austronesians, such as the Ami and Atayal from Taiwan (Mallick et al., 2016), share connections with the Ikawazu Jōmon relative to more inland ancient East Asians (Figure 2B and C), potentially indicating admixture between the Jōmon and southern East Asian populations. Comparisons with nuclear genomes from Fujian in southern China show similar connections (Yang, MA and Fu, Q unpublished observations). Thus, rather than Southeast Asian, the primary signal seems to be associated with Austronesians, who derive from an East Asian population associated with southern China.

Siberian connections, particularly in Hokkaido, have also been a recurring theme of Jōmon-related genetic studies. Several of the mtDNA haplogroups prevalent in the Japanese and Jōmon but rare in mainland East Asians, especially G1b, can be found in southeastern Siberians, highlighting potential contacts along the coast in northern East Asia as well (Sato et al., 2009; Adachi et al., 2011). Pre-modern Ainu show similar mtDNA haplogroups to populations in the Lower Amur region of Siberia and populations belonging to the early historic Okhotsk culture of Hokkaido (600–1200 AD) (Adachi, Kakuda, Takahashi, Kanzawa-Kiriyama, & Shinoda, 2018). Studies of nuclear DNA from Early Neolithic populations in the Primorye region of Russia highlight that the Jōmon are an outgroup to all mainland East Asians, including these more northern populations in Siberia (Sikora et al., 2019; de Barros Damgaard et al., 2018). In the same comparisons set up for coastal southern East Asians above, it can be shown that ancient and present-day coastal northern mainland East Asians dating up to 7700 years ago share connections with the Ikawazu Jōmon relative to more inland East Asians (Figure 2B, C). Ancient northern Siberian ancestry prevalent during the Palaeolithic notable for both its closer relationship with European-related rather than Asian-related ancestry and its impact on Native American ancestry (Raghavan et al., 2014; Sikora et al., 2019) is not found in mainland East Asians or the Jōmon, which emphasizes that the connections are specific to coastal mainland East Asians and the Jōmon.

One explanation for a connection between the Jōmon and coastal East Asians could be that the Jōmon were not completely isolated from mainland East Asians. By 3900 years ago, the date of the oldest Jōmon nuclear genome sampled (from Funadomari, Figure 1), Austronesians were rapidly expanding into islands in the Pacific (Tsang, 1992; Spriggs, 2011). The main patterns observed both in past mtDNA studies and in recent genome-wide studies of the Jōmon all seem to highlight coastal connections, which may suggest that the Jōmon experienced gene flow with populations deriving from mainland East Asia prior to any contact associated with migration of Mumun immigrants from the Korean peninsula. This is supported by archaeological studies that track artefact commonalities resulting from trade and contact along the coast (Bausch, 2017), some of which date back to the Palaeolithic (Morisaki, 2015). This is logical given that the Jōmon were inveterate deep-sea fishers given their harpoon technology, and scores of dugout canoes have been excavated from archaeological sites (Habu, 2010) — although it is not clear how seaworthy they were.

To address the relationship between population movement and the spread of farming in the Japanese archipelago, Jōmon and Mumun ancestry must be well characterized. Here in this section, we have briefly highlighted some key features of Jōmon ancestry using ancient mtDNA and nuclear genomes. First, Jōmon ancestry diverged fairly early from that of mainland East Asians. Second, they do not show notable connections to currently sampled basal Asians, such as the Tianyuan individual or Hòabìnhians. Third, they share coastal connections with coastal populations in northern and southern East Asia. This suggests that one of the major assumptions about the Jōmon may not be true – that they were genetically isolated since the first Palaeolithic migrations to the Japanese archipelago until the Mumun migration at the end of the Jōmon period. If the Jōmon themselves are already partially admixed, then characterizing increased gene flow from the mainland in populations from the Yayoi or more recent historic periods will be substantially more complex.

Advances that utilize rare alleles or long haplotypes (Lawson & Falush, 2012; Schiffels et al., 2016) are increasing the power of demographic analyses focused on more closely related populations, including in Japan (Takeuchi et al., 2017), but these are still difficult to apply in the realm of ancient DNA where obtaining data with high enough coverage is still rare. Present-day genomes are informative on recent history (Takeuchi et al., 2017) but make it difficult to resolve questions about periods as early as the Yayoi. Ancient genomes directly from the Yayoi are needed to clarify early population movement associated with the spread of farming. Such sampling is just starting, where two nuclear genomes from the Shimo-Motoyama rock shelter site in northwest Kyushu dating to the Terminal Yayoi show evidence of admixture between Jōmon- and mainland East Asian-related ancestry, in a region where populations were thought to be of unadmixed Jōmon-lineage (Shinoda et al., 2019). Characterizing the role admixture played during the Yayoi requires understanding of the features of Jōmon ancestry described above to help us understand how to contextualize these and future data.

The remaining parts of this paper focus on populations in the Yayoi to Nara periods, examining (1) how archaeological shifts indicate a complex role for migration and admixture that was probably region-specific and (2) how similarities in dialect types hint at prehistoric migration of admixed Yayoi populations along the Japan Sea coast, which only stresses the need for more data on ancient Yayoi DNA.

The coastal genetic connections proposed are not directly tied to the farmer/language spread hypothesis because they are found in the Jōmon and thus would be prior to the spread of farming. Instead, they are a cautionary point – to understand farmer/language spread, we need to understand who were the pre-existing populations (the Jōmon). If their ancestry is not as genetically isolated as has been argued, then future studies need to account for that.

## Agricultural transition in Japan

In dealing with this problem, first we must define ‘agriculture’. This is not as easy as it appears, since the Jōmon were experienced at plant management, leading to several local domesticated species. Thus, they can be called hunter–gatherer–fisher–horticulturalists. However, the plants that they domesticated or at least managed – soybeans, adzuki, Perilla, sweet chestnuts and Japanese millet (*Echinochloa utilis*) among others – were not suitable for intensive cultivation as carbohydrate sources to become staples (Crawford, 2011; Barnes, 2015: 111–112, and references therein). Crawford would rather discard the distinction between horticulture and agriculture, preferring to deal with proportionalities in ‘resource production’, which includes not only food but other strategic resources such as lacquer and hemp for fibres. Here, we use ‘agriculture’ to refer to the farming of major starch-grain crops: rice (*Oryza sativa*) and millets (*Setaria italica* and *Panicum miliaceum*) in Early Yayoi, with barley and wheat added in later Yayoi.

The Jōmon subsistence system was interrupted by the importation of rice and millets from the Korean Peninsula in the Mumun Period, heralding the start of the Yayoi period in the Japanese Islands. The beginnings of rice agriculture in each region of Japan have been meticulously tracked, first from its source on the continent into North Kyushu (Miyamoto, 2017, 2018, 2019) and then eastwards through the archipelago (Fujio, 2004, 2009, 2013, 2014, 2017). Proto-Japanese speaking Mumun migrants (Whitman, 2011) coming into North Kyushu interacted with Jōmon peoples there, creating an initial admixed Yayoi population which then spread throughout the Japanese islands as they occupied new lands for farming.

**Figure 3.**
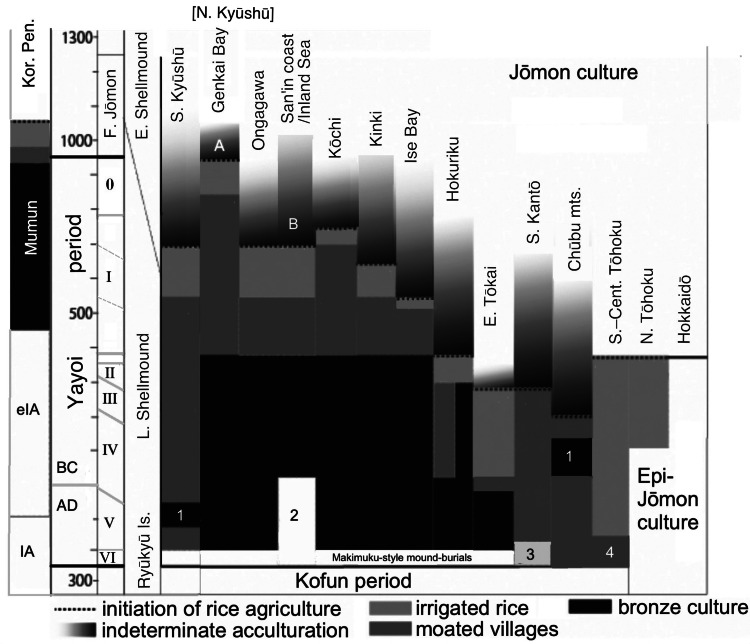
Regional interfaces of expanding agriculture and Jōmon lifeways through phases I–V of the Yayoi period, set against the Mumun and Iron Age periods of the Korean Peninsula and the Shellmound periods of the Ryūkyū Islands. Source: Fujio (2013), fig. 3, modified by GLB with permission. Key: 1, instances of bronze introduction and then rejection; 2, abandonment of bronze ritual use; 3, use of bronze bells; 4, moated burials but not moated villages.

For each region, Fujio has identified a ‘hazy’ period of possible cultivation capped by the clear introduction of rice agriculture (Figure 3); he distinguishes this hazy period, however, from the clear adoption of *irrigated* rice agriculture and the appearance of the continental cultivation toolkit, moated settlements and moated burials. These elements have been treated as the ‘Yayoi package’ (Mizoguchi, 2013) or ‘Yayoi complex’ (Miyamoto, 2016, 2019). Within most of western Honshu Island, Fujio expects that grain cultivation (rice and particularly millets) preceded the formal package. If we assume that the package was instituted by migrating farmers, the hazy periods and non-irrigated rice-farming periods could represent the gradual conversion of Jōmon-lineage peoples to an agricultural way of life. By 380 BC, however, the ‘Yayoi package’ entailing bronze and iron usage was fully established west of the ‘Waist of Honshu’ (between Wakasa Bay and Ise Bay, Figure 1).

Beyond the Waist of Honshu, the uptake of farming was far more diverse. Current research identifies phases of Jōmon pottery usage during the advent of agriculture in the Kinai, Chūbu central mountains, and Kantō regions where cultivation of dry-field crops also preceded irrigated rice agriculture (Shitara & Takashe, 2014; Shitara & Fujio, 2014, pp. 12–13; Takase, 2014; Barnes, 2019). Thus, some farmers in the Yayoi period might have been *neither* genetically admixed with Mumun (being Jōmon-lineage) *nor* Japanese speakers. Rice agriculture was established in Hokuriku and Tōhoku from 380 BC. However, settlements at the northern tip of Honshu failed by 100 BC, with occupants reverting to a hunting and gathering lifestyle. Thus, North Tōhoku is included with Hokkaido in the Epi-Jōmon period, which lasted to 700 AD, but we will refer to Jōmon-lineage people in other parts of Tōhoku also as Epi-Jōmon.

According to Figure 3, irrigated rice agriculture was instituted by 380 BC in Hokuriku and 300 BC in South Kantō, but not until 220 BC in Chūbu and 50 BC in eastern Tōkai. Interestingly, it reached North and South Kantō via different routes. The specific ceramic style accompanying the introduction of rice into Gunma Prefecture in northwest Kantō had its origin near the Japan Sea coast in Niigata Prefecture (Baba, 2008). It most likely arrived there through diffusion along the Japan Sea coast from western Honshu, a completely different route from that arriving in Kanagawa Prefecture along the Pacific seaboard.

North of the Toné River, the essential elements of the Yayoi ‘package’ are mainly missing until ca. 150 AD (Fujio, 2013). Thus, it is assumed that cultivation techniques for rice and millet in South and Central Tōhoku were previously borrowed by Epi-Jōmon peoples living in this area and the Japanese language was spread by later movements of people in the Kofun period.

In the second and third centuries AD, state formation processes began with the widespread adoption of mounded tomb building for those Yayoi elites who had risen to high status within the developing agricultural polities. The succeeding Kofun (‘old tomb’) period (Barnes, 2007) witnessed the establishment of a centralized Yamato state in the fifth-century Kinai region. This was also a century of a second wave of immigration from the Korean Peninsula, stimulated by warfare among Peninsular states. In the fifth and sixth centuries, migrations of elites from western Honshu augmented the Kantō population and introduced equestrian culture.

The Nara period began when a national capital, Heijō (Figure 1), was built in today's Nara Prefecture. At this point, however, the Yamato state was limited to the Kinai region, but alliances were made with outlying chieftains in Kyushu and Kantō. By the mid-seventh century these figures were integrated into a court hierarchy that represented an expanded Yamato state. However, this state did not extend further north than Kantō; peoples who occupied the Tōhoku region were beginning to be called *emishi* in court documents. The Nara state actively pursued military activities to bring this region under court control, although they ultimately failed and the region remained under the rule of prominent *emishi*.

‘*Emishi*’ in the Nara period is understood to have been a word that designated people who lived in the north beyond the reach of the state. It was neither an ethnonym nor referred to a way of life but was an administrative term – and so encompassed everyone living beyond state jurisdiction regardless of subsistence, language, or genetic background. We assume that those *emishi* who practised rice agriculture spoke Japanese, but Ainu place names surviving from Niigata and Fukushima northwards clearly indicate that the Ainu language was being spoken by some *emishi* (see Hudson, 1994, fig. 1). Historical documents record the need for interpreters at least once during the state expansion into the north (the Emishi Wars 774–811), which ended abruptly with the court abandoning its military campaigns (Friday, 1997); the Epi-Jōmon included among the *emishi* were very likely speakers of an Ainu-related language, and most linguists view Ainu as a relic Jōmon language. It is clear that evidence of the Ainu *language* predates the emergence of the Ainu *people* in historical documents in the seventeenth century (Hudson, 1999).

Knowledge of the subsistence system of Tōhoku in the Kofun and Nara periods is not well developed. The Kantō became a prominent horse-breeding area, supplying the Court with horses and cavalry, leading to the development of the medieval samurai (Farris, 1992; Friday, 1997). Further north, Epi-Jōmon, admixed Yayoi descendents and Kofun-period pioneers formed the *emishi* who opposed the Nara Court's efforts at subjugation. By the ninth century, powerful local warlords from southwestern clans such as the Abe and Kiyohara ruled large territories in the north on behalf of the Heian Court, and finally in the twelfth century, the Northern Fujiwara established their rule over Tōhoku. It is significant that, despite the court origins of this family, the coffins of the first three Northern Fujiwara rulers contained only a little rice and no continental millets, but large quantities of barnyard millet (*E. utilis*) (Hudson, 1999, p. 76). This suggests that continental-type agriculture was not a prominent feature of Tōhoku farming even into the Medieval period, with barnyard millet harking back to Jōmon horticultural practices.

## The languages of Japan

As in many other areas of the world, migrations in connection with the spread of agriculture in Japan are thought to have led to large-scale replacement of the languages of indigenous hunter–gatherers (Robbeets & Savelyev, 2017). The only remnant today of the many languages of hunter–gatherers that must have been present in Japan in the Jōmon period is formed by Ainu, an (almost) extinct language on the northeastern island of Hokkaidō. The presence of Ainu place names in Honshū, from Niigata and Fukushima Prefectures northwards, indicates that this region too was once an area where an Ainu-related language was spoken. All other languages in the archipelago now belong to the Japanese language family.

It is likely that at least initially diversification in the language was low, as is often the case when a group of migrants spreads out over a new territory. Dialect borders that later developed in Japan often follow natural barriers such as mountain ranges that impede communication between neighbouring areas. The best-known dialect border for instance, which divides the dialects up into an eastern Japanese and a western Japanese dialect group based on differences in vocabulary and grammar, follows the Hida and Kiso mountain ranges in central Honshū. Most of these differences, for instance those in grammar, do not go back further than the fourteenth century, and many are linked to linguistic influence radiating out from the old capitals of Nara and Kyōto. Some other dialect borders, on the other hand, may have links to the prehistoric migrations that spread agriculture through the islands.

### The unexpected dialect type of the northeast

An example of such an unexpected dialect distribution is the fact that the phonology and tone system of the Tōhoku dialect resemble those of Izumo, on the Japan Sea coast in the west of Honshū, more than those of the adjacent Kantō region.

**Figure 4.**
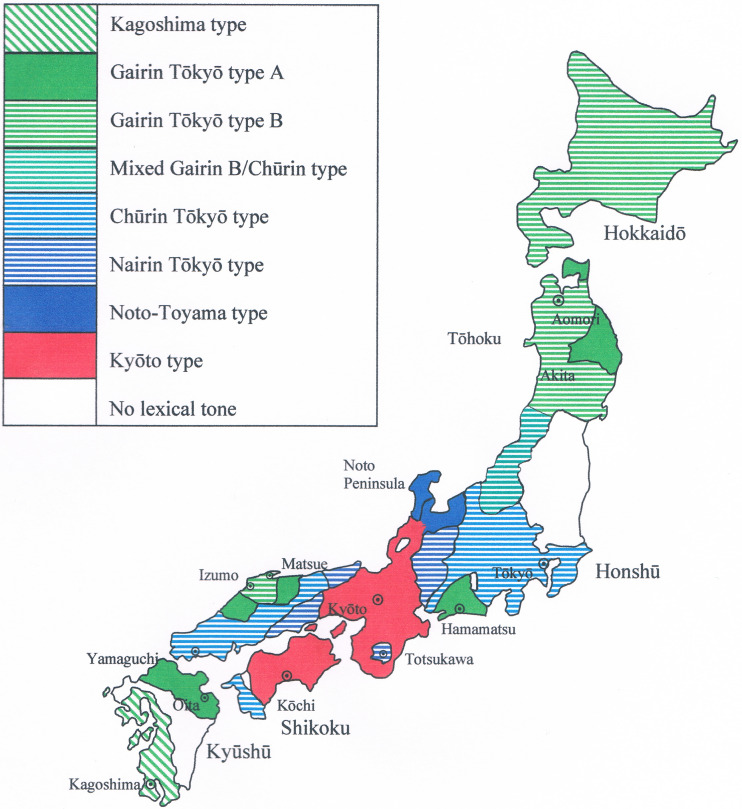
Map of the Japanese tone systems. Adapted by EdB from Wurm and Hattori (1981): no page number.

The tone system of West Kantō, for instance, belongs to the so-called Chūrin or ‘middle circle’ type, in which the division of the words of the language in tone classes is different from that of the Tōhoku region, which has a so-called Gairin or ‘outer circle’ type tone system. These names are derived from the geographical positions of the types relative to each other (Figure 4). An earlier work (De Boer, 2010) analysed the Gairin-type merger pattern of the proto-Japanese tone classes as the result of an innovation that other dialects lacked. As such, a Gairin-type system can develop multiple times independently, as the tonal assimilations that gave rise to the Gairin system are natural (there are, for instance, also two regions with Gairin A tone systems in Kyūshū and Shizuoka), but the agreements between the dialects of Izumo and Tōhoku go deeper.

Within the innovative Gairin type, a further innovation occurred, resulting in a sub-type where high tone shifts away from syllables that contain /i/ or /u/. It can be seen that this type (Gairin B in Figure 4) developed in Izumo in the centre of the region, whereas the older Gairin type (Gairin A) was preserved in the periphery. The Gairin B innovation is also found in the Tōhoku region, except in two areas far removed from the Japan Sea coast. This distribution suggests that the Gairin B innovation was introduced on the Japan Sea coast side, and spread eastward from there.

**Figure 5.**
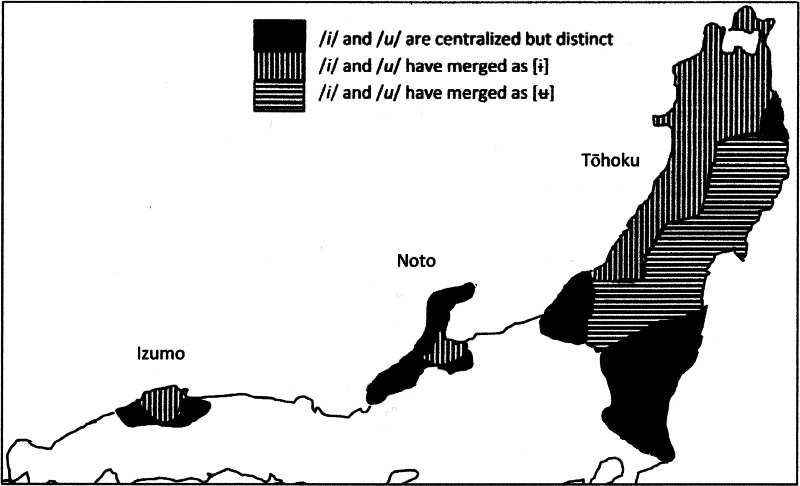
Map of vowel centralization of /i/ and /u/ and merger of /i/ and /u/ after coronal consonants. Adapted by EdB from Kamei, Kōno, and Chino (1989), p. 1760.

The vowel systems of both Izumo and Tōhoku have centralized /i/ and /u/ and raised /e/ and /o/ so that these vowels are all close together and no longer maximally opposed to each other as they are in other Japanese dialects. In central Izumo and in the Tōhoku region (except for a small area far removed from the Japan Sea coast), this has resulted in mergers between vowels (Figure 5). This geographical distribution, too, suggests that an innovation that originated in Izumo was introduced to the northeast by way of the Japan Sea coast.

The East Kantō dialect (Figure S2) takes a somewhat intermediate position between the West Kantō dialect and the Tōhoku dialect. Some centralization of /*i*/ and /*u*/ is present, and the fact that the tonal distinctions have disappeared is most likely the result of a mixture of West Kantō (Chūrin) and Tōhoku (Gairin) influences. From other areas in Japan it is known that confusion between adjacent tone systems with different merger patterns of the tone classes can lead to collapse of the system. The northeastern toneless zone includes not only the East Kantō dialect, but also the Pacific side of southern Tōhoku (Figure 4). On the Japan Sea side of southern Tōhoku the reflexes are mixed Chūrin and Gairin B, but the system has not collapsed.

What does the opposition between the West Kantō tone and vowel system and the northern Tōhoku tone and vowel system and the intermediate zone between them mean for the way in which the northeast was settled by Japanese-speaking farmers? There are a number of things to consider. Vocabulary can be easily borrowed long distance from other dialects, as long as there is contact (for instance through trade along the coast). Phonology, or the sound structure of a language, on the other hand, usually spreads to adjacent areas, with which there is sustained contact. If it spreads to distant areas, migration is the most likely cause, although it is possible for similarities in phonology between far-flung regions to be the result of parallel independent development, if the innovations are easily repeatable and cross-linguistically common.

The similarities between Izumo and Tōhoku (and to a lesser extent the Noto Peninsula) come in shared sets (see Tables S1 and S2; and De Boer, forthcoming), which makes parallel independent development unlikely. It suggests movement of people along the coast in at least two different periods: first from Izumo to the area of the Noto peninsula (present-day Ishikawa and Toyama Prefectures), spreading the set of features listed in Table S1; and later from Izumo to the Tōhoku coast, spreading the set of features listed in Table S2.

### Izumo in the Late Yayoi period

Although Izumo (Shimane Prefecture) is nowadays relatively poor and sparsely populated, this was not always the case. Izumo was one of the most powerful confederacies of the Mid and Late Yayoi periods. It was the great rival of Yamato and the focal point of a wide maritime trade network that included the Japan Sea coast, the Ryūkyūs, Kyushu, Korea and China (Torrance, 2016). Izumo formed alliances with other adjacent regions along the Japan Sea coast; Watanabe (1995) speaks of an ‘Izumo cultural zone’ which he places in the Late Yayoi period.

The corner-projected mound burials typical for Izumo in this period are also found on the Noto Peninsula and in Toyama Prefecture, where they stem from 100–250 AD (Maeda, 2007, p. 6). This makes it likely that the presence of the Izumo vowel system on the Noto Peninsula and in Toyama dates back to the Mid to Late Yayoi period, suggested not only by the congruence of burial types (Figure S3) but also the presence of the Izumo-style vowel mergers in Toyama (Figure 5). The Izumo-style tone systems (Gairin A and Gairin B) had apparently not yet developed, as the tone system of the Noto Peninsula does not share these innovations (Figure 4). Some other innovations have occurred in the local tone systems since then, but not the same as those shared by Izumo and Tōhoku.

The fact that the Izumo tonal innovations are today present in the northern Tōhoku region, and in a mixed form along the southwestern Tōhoku coast, means that migrations from Izumo to these areas must have taken place after the Gairin B innovations developed. The presence of both Gairin A and Gairin B in the northeast may indicate migrations in different periods. It may also be related to different points of departure from Izumo, as political and economic prominence in Izumo fluctuated historically between the eastern and western parts (Piggott, 1989; Torrance, 2016). The Gairin B tonal innovation is most advanced in western Izumo (Hirako, 2017), meaning that it most likely started there and only gradually spread to eastern Izumo. In the Tōhoku region too, the Gairin B system was most likely present in a smaller area in earlier times. The tone shifts typical of this system continue to spread to adjacent areas: recent fieldwork (Boiko, 2018) has shown that, by now, the Gairin A area on the Shimokita Peninsula has disappeared, and instead, the Gairin B tone shifts have been adopted.

### Looking for the time of the migrations

Exploring the similarity of the Izumo and Tōhoku dialects, it is necessary to examine opportunities for migration of Izumo dialect speakers into the northeast. Although the very first forays of rice farmers from western Japan to the northern tip of Honshū already took place in the Middle Yayoi period, these settlements were later abandoned. The full ‘Yayoi package’ reached the coastal areas of Ishikawa, Toyama and western Niigata as early as 300 BC. It is possible that rice farmers settling along the coast departed from Izumo, but there is no direct evidence for this. Clear evidence of influence from Izumo is more recent: the Izumo-style corner-projected burial mounds in Ishikawa and Toyama date from 100–250 AD. While the full Yayoi ‘package’ appeared briefly in the central mountains (Figure 3: #1, Chūbu district), it also intruded into western Gunma Prefecture at this time. The ceramics accompanying the irrigated-rice culture package derived from the Jōetsu Basin in western Niigata and spread through the river valleys of Nagano Prefecture into western Gunma (Baba, 2008).

It is unlikely that the rice farmers who moved inland from western Niigata spoke a dialect with the features typical of Izumo. Linguistic influence from Izumo in the form of migration was not only later, it also may have been initially limited to Ishikawa (i.e. the Noto Peninsula) and Toyama, as Izumo-style mound burials have not been found in Niigata. If there was already some Izumo influence on the dialect of Niigata at the time when rice farmers moved inland to Gunma, it would have been on the vowel system only. The tonal innovations in the Izumo dialect had not yet occurred, judging from the fact that these are not present in the dialects of Ishikawa and Toyama. What applies to the dialect of Niigata also applies to Nagano (Chūbu) and Gunma (West Kantō), which was settled from Niigata, and to Tochigi and Ibaraki (East Kantō), which were all settled overland. If the Tōhoku region was entirely settled overland from these areas, the similarities with the Izumo dialect remain unexplained.

Can migration of Izumo dialect speakers into Tōhoku in later periods explain the similarities between the dialects? Piggott (1989) examined the history of Izumo throughout the Late Yayoi and Kofun periods, distinguishing between eastern and western Izumo. Izumo's unique mound-burial culture of Late Yayoi gave way to cultural and political inroads from Kibi to the south and Yamato itself, first into western Izumo. The chieftains of eastern Izumo maintained trade relations with northern Koshi (western Niigata) into the sixth century, but by the 540s the Izumo chieftains had all allied with Yamato (Piggott, 1989, pp. 59–60). Torrance (2016, p. 4), on the other hand, argues that Izumo remained an important and independent presence along the Japan Sea coast, at least until the late sixth or early seventh century.

Under the circumstances it is possible that groups from Izumo, avoiding Yamato pressure, would migrate northward along the Japan Sea coast. Diffusion into Tōhoku through mountain basins and over passes could have taken place, but were the numbers enough for linguistic change throughout Tōhoku? The mixed nature (and collapse) of the tone systems in south and central Tōhoku suggest that there was some influence but not enough to determine the dialect. The linguistic influence in northern Tōhoku, however, was much stronger.

Hudson (2017) mentions that until the fifth century the pottery types of the northern Tōhoku region and Hokkaidō were identical. From the fifth to the seventh centuries, however, this pottery type starts to disappear from northern Tōhoku, and is from then on restricted to Hokkaidō only. This development has been taken to mean that the Epi-Jōmon population among the *emishi* who, from the evidence of place-names, must have spoken an Ainu-related language, moved away from northern Honshū into Hokkaidō in that period. According to Matsumoto (2018, p. 158), after the late fifth century there are no traces of habitation in the northern Tōhoku for about a century, after which a new population arrives in the late sixth century. The new arrivals were archeologically indistinguishable from Kofun cultures elsewhere in Japan (Matsumoto, 2018, p. 159).

If the people arriving in the late sixth century were from Izumo, then northern Tōhoku would have been sparsely populated at that time, as the Epi-Jōmon population had for a large part moved away. That means that the new population could rapidly spread out over the entire area, which would explain why the Izumo-style tone system was preserved well there, in contrast to the situation in south and central Tōhoku. In south and central Tōhoku there was interference not only from other dialects but most likely also from languages of the local Epi-Jomon population, who were no longer hunter–gatherers but settled farmers by then. The relatively low internal diversity of the Tōhoku dialects may be attributed to the overall later spread of Japanese to the northeast compared with other areas of mainland Japan (Inoue, 1992).

Migration of people from Izumo to the northeast may even have left a genetic trace. Saitō (2017, p. 127) uses a principal component analysis (PCA), in which nuclear DNA from modern Tōhoku individuals is compared with nuclear DNA from modern Ainu, Ryūkyūan and mainland Japanese individuals (Figure S4). A PCA simplifies complex datasets, creating summaries that emphasize the components explaining the maximum amount of variance. In population genetics, this method is often a quick and useful tool to examine population relationships, as the maximum amount of variance is often attributed to shared population history. However, it is not a formal test of shared ancestry.

Saitō concludes that, in contrast to Ryūkyūans, the Tōhoku population has very low traces of Ainu-related ancestry, which was used as a proxy for Jōmon-related ancestry. He also includes a PCA showing the position of Izumo individuals relative to Okinawan and Kantō individuals (Saitō, 2017, p. 155), and he remarks upon the fact that these individuals occupy the same relative position as the Tōhoku individuals (Figure S4), namely they are to the lower right of the average Mainland Japanese individual.

It is better to be cautious in drawing conclusions until more detailed comparisons from different areas of Japan are available, but the initial results are definitely interesting in light of the dialectal similarities between Izumo and the Tōhoku region.

## Concluding remarks

Confronted with the Yayoi farmers and their new mode of subsistence, the different ethnic groups that were already present in Japan could react in different ways: assimilation and adoption of the new techniques, specialization in products with which to trade with the new farming populations, resistance or withdrawal. The available options differed per region and per period, influencing the way in which the Japanese language spread.

The present-day dialect distribution in northern Honshū suggests that a considerable part of the early historic *emishi* population were speakers of an Izumo-type dialect. There certainly will have been Epi-Jomon people speaking an Ainu-related language among the *emishi* ranks, but what seems clear is that the Izumo-type dialect of Tōhoku must have already had a strong foothold in the northeast before the period of the Emishi Wars of the late eighth century. That dialect is proposed to have spread either with the introduction of rice agriculture by admixed Yayoi peoples in Mid to Late Yayoi, or in the expansion of the mounded tomb culture in the Kofun period.

This study has highlighted a strong need to bring Japanese scholarship on the history and archaeology of Tōhoku into consideration with the farming/language dispersal hypothesis. The complexity of population movements in this area is illustrated by Hakomori (2013). Also needed is ancient DNA sequencing at fine temporal and geographic scales. With increasingly sophisticated techniques for quantifying genetic admixtures (cf. Chikhi, Nichols, Barbujani, & Beaumont, 2002; Dupanloup, Bertorelle, Chikhi, & Barbujani, 2004; Wollstein & Lao 2015), maybe Yayoi- to Nara-period skeletal material from the Kantō and Tōhoku regions will enlighten us on the interactions between indigenous peoples and migrants in the spread of agriculture to the northeast. The deep ancestry associated with the Jōmon will aid in highlighting changing proportions of different types of Asian-related ancestry in these different regions, although it behooves us to be alert to Jōmon continental interaction prior to the Yayoi period.

## Supplementary material

For supplementary material accompanying this paper visit http://dx.doi.org/10.1017/ehs.2020.7. click here to view supplementary material

## Research transparency and reproducibility

The genetic data shown are all previously published, as described in Table S5. Data in Table S5 are presented in a format that can be read by the software Admixtools (version 412; Patterson et al., 2012) where genotyped data are available for the ‘1240K’ SNP panel and can be found in a publicly available database: v42.4, David Reich Lab Harvard University (Reich, 2020). Data used to visualize Figures 2 and S1 are reproduced in Tables S3 and S4. Scripts used to conduct the genetic analyses and visualize the data can be made available upon request to myang@richmond.edu

## References

[ref1] Adachi, N., Shinoda, K., Umetsu, K., & Matsumura, H. (2009). Mitochondrial DNA analysis of Jomon skeletons from the Funadomari site, Hokkaido, and its implication for the origins of Native Americans. American Journal of Physical Anthropology, 138(3), 255–265. 10.1002/ajpa.20923 18951391

[ref2] Adachi, N., Shinoda, K. I., Umetsu, K., Kitano, T., Matsumura, H., Fujiyama, R., … Tanaka, M. (2011). Mitochondrial DNA analysis of Hokkaido Jomon skeletons: remnants of archaic maternal lineages at the southwestern edge of former Beringia. American Journal of Physical Anthropology, 146(3), 346–360. 10.1002/ajpa.21561 21953438

[ref3] Adachi, N., Kakuda, T., Takahashi, R., Kanzawa-Kiriyama, H., & Shinoda, K. (2018). Ethnic derivation of the Ainu inferred from ancient mitochondrial DNA data. American Journal of Physical Anthropology, 165(1), 139–148. 10.1002/ajpa.23338 29023628 PMC5765509

[ref4] Baba, S. (2008). Reconsidering the chronology of pottery styles of Mid-Yayoi, and theories of distribution: putting into perspective the possibility of Yayoi Period trading. Bulletin of the National Museum of Japanese History, 145, 101–174 (in Japanese with English title and summary).

[ref5] Barnes, G. L. (2007). State Formation in Japan: Emergence of a 4th-century Ruling Elite. London: Routledge.

[ref6] Barnes, G. L. (2015). Archaeology of East Asia: the Rise of Civilization in China, Korea, and Japan. Oxford: Oxbow Books.

[ref7] Barnes, G. L. (2019). The Jōmon–Yayoi transition in eastern Japan: enquiries from the Kantō Region. Japanese Journal of Archaeology, 7, 1–51.

[ref8] Bausch, I. (2017). Prehistoric networks across the Korea Strait (5000–1000 BCE). In T. Hodos (Ed.), The Routledge Handbook of Archaeology and Globalization (pp. 413–433). Abingdon: Routledge.

[ref9] Bellwood, P., & Renfrew, C. (Eds.) (2002). Examining the Farming/Language Dispersal Hypothesis. Cambridge: The McDonald Institute.

[ref10] Boiko, M. (2018). What can the Tōkyō Gairin dialects tell us about the history of Japanese pitch accent? Poster presentation, 5th International Conference on Phonetics and Phonology (NINJAL ICPP 2018), National Institute for Japanese Language and Linguistics, Tōkyō, 26–28 October.

[ref11] Chikhi, L., Nichols, R. A., Barbujani, G., & Beaumont, M. A. (2002). Y genetic data support the Neolithic demic diffusion model. Proceedings of the National Academy of Sciences, 99, 11008–11013. 10.1073/pnas.162158799 PMC123201 12167671

[ref12] Crawford, G. W. (2011). Advances in understanding early agriculture in Japan. Current Anthropology, 52(4), S331–S345. Retrieved from www.jstor.org/stable/10.1086/658369.

[ref13] de Barros Damgaard, P., Martiniano, R., Kamm, J., Moreno-Mayar, J. V., Kroonen, G., Peyrot, M., … Willerslev, E. (2018). The first horse herders and the impact of early Bronze Age steppe expansions into Asia. Science, 360(6396). 10.1126/science.aar7711. PMC6748862 29743352

[ref14] De Boer, E. M. (forthcoming) The classification of the Japonic languages. In M. Robbeets (Ed.), Oxford Guide to the Transeurasian Languages. Oxford: Oxford University Press.

[ref15] De Boer, E. M. (2010). The Historical Development of Japanese Tone. Part 1 From Proto-Japanese to the Modern Dialects. Part 2 The Introduction and Adaptation of the Middle Chinese Tones in Japan. Wiesbaden: Harrassowitz.

[ref16] De Boer, E. M. (2017). Review of *Handbook of the Ryukyuan languages: History, structure and use*. Studies in Language, 41(1), 781–790.

[ref17] Dupanloup, I., Bertorelle, G., Chikhi, L., & Barbujani, G. (2004). Estimating the impact of prehistoric admixture on the genome of Europeans. Molecular Biology and Evolution, 21, 1361–1372. 15044595 10.1093/molbev/msh135

[ref18] Farris, W. W. (1992). Heavenly Warriors: the evolution of Japan's military, 500-1300. Cambridge, MA: Harvard University Press.

[ref19] Friday, K. F. (1997). Pushing beyond the pale: The Yamato conquest of the Emishi and northern Japan. The Journal of Japanese Studies, 23(1), 1–24.

[ref20] Fu, Q., Meyer, M., Gao, X., Stenzel, U., Burbano, H. A., Kelso, J., & Paabo, S. (2013). DNA analysis of an early modern human from Tianyuan Cave, China. Proceedings of the National Academy of Sciences USA, 110(6), 2223–2227. 10.1073/pnas.1221359110 PMC3568306 23341637

[ref21] Fujio, S. (2004). Nihon rettō ni okeru nōkō no hajimari [The beginning of agriculture in the Japanese Islands]. In K. Kenkyūkai (Ed.), Cultural Diversity and the Archaeology of the 21st Century (pp. 62–73). Okayama: The Society of Archaeological Studies (in Japanese).

[ref22] Fujio, S. (2009). Interaction between the Jomon farmer and the Yayoi farmer in the beginning of the Yayoi Period along the Old Kawachi Lake. Bulletin of the National Museum of Japanese History, 152, 373–400 (in Japanese with English title and summary).

[ref23] Fujio, S. (2013). The frame of the Yayoi culture: Is wet rice cultivation with irrigation system an indicator of the Yayoi culture? Bulletin of the National Museum of Japanese History, 178, 85–120 (in Japanese with English title and summary).

[ref24] Fujio, S. (2014). When did the wet rice cultivation with the irrigation system begin in the Western Japan. Bulletin of the National Museum of Japanese History, 183, 113–143 (in Japanese with English title and abstract).

[ref25] Fujio, S. (2017). How should we view the Yayoi culture of Tōhoku? Presentation at the symposium ‘Didn't Yayoi Culture Exist in Sendai Plain?: Lecture and Discussion on Mr. Shinichiro Fujio's New Theory’, 19–20 November 2017, Sendai (in Japanese).

[ref26] Gakuhari, T., Nakagome, S., Rasmussen, S., Allentoft, M., Sato, T., Korneliussen, T., … Oota, H. (2019). Jomon genome sheds light on East Asian population history. bioRxiv, 579177. 10.1101/579177 PMC7447786 32843717

[ref27] Haber, M., Mezzavilla, M., Xue, Y., & Tyler-Smith, C. (2016). Ancient DNA and the rewriting of human history: be sparing with Occam's razor. Genome Biology, 17(1), article 1. 10.1186/s13059-015-0866-z 26753840 PMC4707776

[ref28] Habu, J. (2004). Ancient Jomon of Japan. Cambridge: Cambridge University Press.

[ref29] Habu, J. (2010). Seafaring and the development of cultural complexity in Northeast Asia: evidence from the Japanese archipelago. In A. Anderson, J. H. Barrett, & K. V. Boyle (Eds.), Global Origins and the Development of Seafaring (pp. 159–170). Cambridge: McDonald Institute for Archaeological Research.

[ref30] Hakomori, K. (2013). Tohoku Kofun population: Sixth through eighth centuries AD. Emishi-ezo.net. Retrieved from http://emishi-ezo.net/Tohoku%20kofun%20population.htm

[ref31] Hammer, M. F., & Horai, S. (1995). Y-Chromosomal DNA variation and the peopling of Japan. American Journal of Human Genetics, 56(4), 951–962. Retrieved from https://www.ncbi.nlm.nih.gov/pmc/articles/PMC1801189/7717406 PMC1801189

[ref32] Hammer, M. F., Karafet, T. M., Park, H., Omoto, K., Harihara, S., Stoneking, M., & Horai, S. (2006). Dual origins of the Japanese: Common ground for hunter–gatherer and farmer Y chromosomes. Journal of Human Genetics, 51(1), 47–58. 10.1007/s10038-005-0322-0 16328082

[ref33] Hanihara, K. (1991). Dual structure model for the population history of the Japanese. Japan Review, 2, 1–33. Retrieved from http://www.jstor.org/stable/25790895

[ref34] Hanihara, K. (2000). The dual structure model: a decade since its first proposal. In K. Omoto (Ed.), Newsletter Special Issue: Interdisciplinary Study on the Origin of Japanese Peoples and Cultures (p. 4). Kyoto: Nichibunken.

[ref35] Hirako, T. (2017). Izumo chiiki nana hōgen no meishi akusento shiryō: 1–3 mora go [Materials on the accent of nouns in 7 dialects in the Izumo region: Words of 1–3 moras]. Jissen Koku-bungaku, 91, 320–378 (in Japanese).

[ref36] Hong, S. B., Kim, K. C., & Kim, W. (2014). Mitochondrial DNA haplogroups and homogeneity in the Korean population. Genes and Genomics, 36(5), 583–590. 10.1007/s13258-014-0194-9

[ref37] Horai, S., Hayasaka, K., Murayama, K., Wate, N., Koike, H., & Nakai, N. (1989). DNA amplification from ancient human skeletal remains and their sequence analysis. *Proceedings of the Japan Academy*, Series B, 65(10), 229–233. 10.2183/pjab.65.229

[ref38] Hudson, M. (1994). The linguistic prehistory of Japan: some archaeological speculations. Anthropological Science, 102(3), 231–255.

[ref39] Hudson, M. J. (1999). Ainu ethnogenesis and the Northern Fujiwara. Arctic Anthropology, 36(1/2): 73–83.

[ref40] Hudson, M. J. (2017). Okhotsk and Sushen: history and diversity in Iron Age maritime hunter–gatherers of northern Japan. In B. Finlayson, & G. Warren (Eds.), The Diversity of Hunter Gatherer Pasts (pp. 68–78). Oxford: Oxbow Books.

[ref41] Igawa, K., Manabe, Y., Oyamada, J., Kitagawa, Y., Kato, K., Ikematsu, K., … Rokutanda, A. (2009). Mitochondrial DNA analysis of Yayoi period human skeletal remains from the Doigahama site. Journal of Human Genetics, 54(10), 581–588 (Japanese and English versions). 10.1038/jhg.2009.81 19696790

[ref42] Inoue, F. (1992). Hōgen no tayōsei to Nihon bunka no nagare [Dialectal diversity and the course of Japanese culture]. Nihongo-gaku, 11, 57–67.

[ref43] ISOGG (International Society of Genetic Genealogy). (2019). Y-DNA haplogroup tree 2019, Version 14.177, 8 October. Retrieved from http://www.isogg.org/tree/ (21 October 2019).

[ref44] Jeong, C., Nakagome, S., & Di Rienzo, A. (2016a). Deep history of East Asian populations revealed through genetic analysis of the Ainu. Genetics, 202(1), 261–272. 10.1534/genetics.115.178673 26500257 PMC4701090

[ref45] Jeong, C., Ozga, A. T., Witonsky, D. B., Malmstrom, H., Edlund, H., Hofman, C. A., … Warinner, C. (2016b). Long-term genetic stability and a high-altitude East Asian origin for the peoples of the high valleys of the Himalayas arc. Proceedings of the National Academy of Sciences USA, 113(27), 7485–7490. 10.1073/pnas.1520844113 PMC4941446 27325755

[ref46] Jeong, C., Wilkin, S., Amgalantugs, T., Bouwman, A. S., Taylor, W. T. T., Hagan, R. W., … Warinner, C. (2018). Bronze Age population dynamics and the rise of dairy pastoralism on the eastern Eurasian steppe. Proceedings of the National Academy of Sciences, 115(48), E11248–E11255. 10.1073/pnas.1813608115 PMC6275519 30397125

[ref47] Jinam, T. A., Kanzawa-Kiriyama, H., Inoue, I., Tokunaga, K., Omoto, K., & Saitou, N. (2015). Unique characteristics of the Ainu population in Northern Japan. Journal of Human Genetics, 60(10), 565–571. 10.1038/jhg.2015.79 26178428

[ref48] Kamei, T., Kōno, R., & Chino, E. (Eds.) (1989). Gengo-gaku dai-jiten v. 2, Sekai gengo-hen [Dictionary of Linguistcs, v. 2, The languages of the world]. Tōkyō: Sansei-dō (in Japanese).

[ref49] Kanzawa-Kiriyama, H., Saso, A., Suwa, G., & Saitou, N. (2013). Ancient mitochondrial DNA sequences of Jomon teeth samples from Sanganji, Tohoku district, Japan. Anthropological Science, 121(2), 89–103. 10.1537/ase.121113

[ref50] Kanzawa-Kiriyama, H., Kryukov, K., Jinam, T. A., Hosomichi, K., Saso, A., Suwa, G., … Saitou, N. (2017). A partial nuclear genome of the Jomons who lived 3000 years ago in Fukushima, Japan. Journal of Human Genetics, 62(2), 213–221. doi:10.1038/jhg.2016.110 27581845 PMC5285490

[ref51] Kanzawa-Kiriyama, H., Jinam, T. A., Kawai, Y., Sato, T., Hosomichi, K., Tajima, A., … Shinoda, K.-I. (2019). Late Jomon male and female genome sequences from the Funadomari site in Hokkaido, Japan. Anthropological Science, advpub. 10.1537/ase.190415

[ref52] Ae-Jin, K., Kijeong, K., Jee-Hye, C., Eun-Ha, C., Yu-Jin, J., Na Young, M., … Keun-Cheol, K. (2010). Mitochondrial DNA analysis of ancient human bones excavated from Nukdo island, S. Korea. BMB Reports, 43(2), 133–139. 20193133 10.5483/bmbrep.2010.43.2.133

[ref53] Kivisild, T., Tolk, H.-V., Parik, J., Wang, Y., Papiha, S. S., Bandelt, H.-J., & Villems, R. (2002). The emerging limbs and twigs of the East Asian mtDNA tree. Molecular Biology and Evolution, 19(10), 1737–1751. 10.1093/oxfordjournals.molbev.a003996 12270900

[ref54] Ko, A. M.-S., Chen, C.-Y., Fu, Q., Delfin, F., Li, M., Chiu, H.-L., … Ko, Y.-C. (2014). Early Austronesians: Into and out of Taiwan. American Journal of Human Genetics, 94(3), 426–436. 10.1016/j.ajhg.2014.02.003 24607387 PMC3951936

[ref55] Krings, M., Stone, A., Schmitz, R. W., Krainitzki, H., Stoneking, M., & Pääbo, S. (1997). Neandertal DNA sequences and the origin of modern humans. Cell, 90(1), 19–30. 10.1016/S0092-8674(00)80310-4 9230299

[ref56] Lan, Q., Xie, T., Jin, X., Fang, Y., Mei, S., Yang, G., & Zhu, B. (2019). MtDNA polymorphism analyses in the Chinese Mongolian group: Efficiency evaluation and further matrilineal genetic structure exploration. Molecular Genetics and Genomic Medicine, 7(10), e934. 10.1002/mgg3.934 PMC6785450 31478599

[ref57] Lawson, D. J., & Falush, D. (2012). Population identification using genetic data. Annual Review of Genomics and Human Genetics, 13(1), 337–361. 10.1146/annurev-genom-082410-101510 22703172

[ref58] Lipson, M., Cheronet, O., Mallick, S., Rohland, N., Oxenham, M., Pietrusewsky, M., … Reich, D. (2018). Ancient genomes document multiple waves of migration in Southeast Asian prehistory. Science, 361(6397), 92–95. 10.1126/science.aat3188 29773666 PMC6476732

[ref59] Maeda, K. (2007). Koshi no yosumi tosshutsugata funkyūbo. [The corner-projected burial mounds of Koshi.] Kana-dai Kōko, 57, 5–12.

[ref60] Mallick, S., Li, H., Lipson, M., Mathieson, I., Gymrek, M., Racimo, F., … Reich, D. (2016). The Simons Genome Diversity Project: 300 genomes from 142 diverse populations. Nature, 538(7624), 201–206. 10.1038/nature18964 27654912 PMC5161557

[ref61] Matsumoto, T. (2018). Tsukurareta Emishi. [*The Manufactured Emishi*.] Tōkyō: Dōseisha (in Japanese).

[ref62] McColl, H., Racimo, F., Vinner, L., Demeter, F., Gakuhari, T., Moreno-Mayar, J. V., … Willerslev, E. (2018). The prehistoric peopling of Southeast Asia. Science, 361(6397), 88–92. 10.1126/science.aat3628 29976827

[ref63] Miyamoto, K. (2016). Archaeological explanation for the diffusion theory of the Japonic and Koreanic Languages. Japanese Journal of Archaeology, 4, 53–75.

[ref64] Miyamoto, K. (2017). The beginnings of farming in Northeast Asia and Yayoi origins. Tokyo: Dōseisha (in Japanese).

[ref65] Miyamoto, K. (2018). Re-examining the absolute date of Yayoi-period beginnings. Kōkogaku Zasshi, 100(2), 1–27 (in Japanese).

[ref66] Miyamoto, K. (2019). The spread of rice agriculture during the Yayoi Period: from the Shandong Peninsula to the Japanese Archipelago via the Korean Peninsula. Japanese Journal of Archaeology, 6(2), 109–124.

[ref67] Mizoguchi, K. (2013). The Archaeology of Japan: From the Earliest Rice Farming Villages to the Rise of the State. Cambridge: Cambridge University Press.

[ref68] Moreno-Mayar, J. V., Potter, BA., Vinner, L., Steinrücken, M., Rasmussen, S., Terhorst, J., … Willerslev, E. (2018). Terminal Pleistocene Alaskan genome reveals first founding population of Native Americans. Nature, 553, 203. 10.1038/nature25173, and https://www.nature.com/articles/nature25173#supplementary-information 29323294

[ref69] Morisaki, K. (2015). Appearance of *hakuhen-sentoki* (HS points) and second modern human migration into Japan. In Y. Kaifu, M. Izuho, T. Goebel, H. Sato, & A. Ono (Eds.), Emergence and Diversity of Modern Human Behavior in Paleolithic Asia (pp. 376–388). College Station, TX: Texas A&M University Press.

[ref70] Nakagome, S., Sato, T., Ishida, H., Hanihara, T., Yamaguchi, T., Kimura, R., … Consortium, ADR. (2015). Model-based verification of hypotheses on the origin of modern Japanese revisited by Bayesian inference based on genome-wide SNP data. Molecular Biology and Evolution, 32(6), 1533–1543. 10.1093/molbev/msv045 25758010

[ref71] Nasu, H., & Momohara, A. (2016). The beginnings of rice and millet agriculture in prehistoric Japan. Quaternary International, 397, 504–512 (in Japanese).

[ref72] Nordborg, M. (1998). On the probability of Neanderthal ancestry. The American Journal of Human Genetics, 63(4), 1237–1240. 10.1086/302052 9758610 PMC1377484

[ref73] Oota, H., Saitou, N., Matsushita, T., & Ueda, S. (1995). A genetic study of 2,000-year-old human remains from Japan using mitochondrial DNA sequences. American Journal of Physical Anthropology, 98, 133–145. 8644875 10.1002/ajpa.1330980204

[ref74] Patterson, N., Moorjani, P., Luo, Y., Mallick, S., Rohland, N., Zhan, Y., … Reich, D. (2012). Ancient admixture in human history. Genetics, 192(3), 1065–1093. 10.1534/genetics.112.145037 22960212 PMC3522152

[ref75] Piggott, J. (1989). Sacral kingship and confederacy in early Izumo. Monumenta Nipponica, 44(1), 45–74.

[ref76] Raghavan, M., Skoglund, P., Graf, K. E., Metspalu, M., Albrechtsen, A., Moltke, I., … Willerslev, E. (2014). Upper Palaeolithic Siberian genome reveals dual ancestry of Native Americans. Nature, 505(7481), 87–91. 10.1038/nature12736 24256729 PMC4105016

[ref77] Reich, D. (2020). Downloadable genotypes of present-day and ancient DNA data (compiled from published papers), v42.4. David Reich Laboratory Harvard University. Retrieved from https://reich.hms.harvard.edu/downloadable-genotypes-present-day-and-ancient-dna-data-compiled-published-papers

[ref78] Renfrew, C. (1987). Archaeology and Language: the Puzzle of Indo-European Origin. London: Jonathan Cape.

[ref79] Robbeets, M. (2005). Is Japanese related to Korean, Tungusic, Mongolic and Turkic? Turcologica Herausgegeben von Lars Johanson, vol. 64. Wiesbaden: Harrassowitz Verlag.

[ref80] Robbeets, M. (2017). Austronesian influence and Transeurasian ancestry in Japanese: a case of farming/language dispersal. Language Dynamics and Change, 7, 210–251.

[ref81] Robbeets, M., & Savelyev, A. (Eds.) (2017). Language Dispersal Beyond Farming. Amsterdam: John Benjamins.

[ref82] Rosenberg, N. A., & Nordborg, M. (2002). Genealogical trees, coalescent theory and the analysis of genetic polymorphisms. Nature Reviews Genetics, 3(5), 380–390. 10.1038/nrg795 11988763

[ref83] Saitō, N. (2017). Kaku DNA kaiseki de tadoru Nihonjin no genryū. [Tracing the origin of the Japanese through the analysis of nuclear DNA.] Tōkyō: Kawade shobō (in Japanese).

[ref84] Sato, T., Amano, T., Ono, H., Ishida, H., Kodera, H., Matsumara, H., … Masuda, R. (2009). Mitochondrial DNA haplogrouping of the Okhotsk people based on analysis of ancient DNA: An intermediate of gene flow from the continental Sakhalin people to the Ainu. Anthropological Science, 117(3), 171–180.

[ref85] Schiffels, S., Haak, W., Paajanen, P., Llamas, B., Popescu, E., Loe, L., … Durbin, R. (2016). Iron Age and Anglo-Saxon genomes from East England reveal British migration history. Nature Communications, 7, 10408. 10.1038/ncomms10408. Retrieved from https://www.nature.com/articles/ncomms10408#supplementary-information PMC4735688 26783965

[ref86] Schmidt, R., & Seguchi, N. (2014). Jomon Culture and the peopling of the Japanese archipelago: Advancements in the fields of morphometrics and ancient DNA. Japanese Journal of Archaeology, 2(1), 34–59. Retrieved from http://www.jjarchaeology.jp/contents/archives/jja_2014_01.html

[ref87] Shinoda, K., Kamisawa, H., Kakuda, T., & Adachi, N. (2019). Special genetic characteristics of northwest Kyushu Yayoi: Analysis of the core genome of skeletal materials from the Shimo–Motoyama rock shelter site in Sasebo City. Anthropological Science, 127(1), 25–43.

[ref88] Shitara, H., & Fujio, S. (2014). Ceramic chronology chart. In Rekihaku (Ed.), What is the Yayoi? (pp. 12–13). Sakura: National Museum for Japanese History.

[ref89] Shitara, H., & Takashe, K. (2014). The beginning of the cereal cultivation in the south-west Kanto district by the analysis of replica. Bulletin of the National Museum of Japanese History, 185, 511–530 (in Japanese with English title and summary).

[ref90] Sikora, M., Pitulko, V. V., Sousa, V. C., Allentoft, M. E., Vinner, L., Rasmussen, S., … Willerslev, E. (2019). The population history of northeastern Siberia since the Pleistocene. Nature, 570, 182–188. 10.1038/s41586-019-1279-z 31168093 PMC7617447

[ref91] Skoglund, P., Posth, C., Sirak, K., Spriggs, M., Valentin, F., Bedford, S., … Reich, D. (2016). Genomic insights into the peopling of the Southwest Pacific. Nature, 538(7626), 510–513. 10.1038/nature19844 27698418 PMC5515717

[ref92] Spriggs, M. (2011). Archaeology and the Austronesian expansion: where are we now? Antiquity, 85, 510–538.

[ref93] Tajima, A., Hayami, M., Tokunaga, K., Juji, T., Matsuo, M., Marzuki, S., … Horai, S. (2004). Genetic origins of the Ainu inferred from combined DNA analyses of maternal and paternal lineages. Journal of Human Genetics, 49, 187–193. 10.1007/s10038-004-0131-x 14997363

[ref94] Takase, K. (2014). Settlements with and without paddy fields. In Rekihaku (Ed.), Exhibition Catalog: What is ‘Yayoi’ (p. 101). Sakura: National Museum for Japanese History (in Japanese).

[ref95] Takeuchi, F., Katsuya, T., Kimura, R., Nabika, T., Isomura, M., Ohkubo, T., … Kato, N. (2017). The fine-scale genetic structure and evolution of the Japanese population. PLoS One, 12(11), e0185487. 10.1371/journal.pone.0185487 PMC5665431 29091727

[ref96] Tanaka, M., Cabrera, V. M., Gonzalez, A. M., Larruga, J. M., Takeyasu, T., Fuku, N., … Shimodaira, H. (2004). Mitochondrial genome variation in Eastern Asia and the peopling of Japan. Genome Research, 14(10a), 1832–1850. 10.1101/gr.2286304 15466285 PMC524407

[ref97] Torrance, R. (2016). The infrastructure of the gods: Izumo in the Yayoi and Kofun periods. Japan Review, 29, 3–38.

[ref98] Tsang, C.-H. (1992). Archaeology of the Peng-hu Islands. Taipei: Academia Sinica.

[ref99] Watanabe, S. (1995). Izumo rengō: Seiritsu to saihen. [The Izumo alliance: Its formation and reorganization.] In Y. Takioto (Ed.), Izumo sekai to kodai no San'in [The world of Izumo and the ancient San'in region] (pp. 61–84). Tokyo: Meicho Shuppan (in Japanese).

[ref100] Watanabe, Y., Naka, I., Khor, S. S., Sawai, H., Hitomi, Y., Tokunaga, K., & Ohashi, J. (2019). Analysis of whole Y-chromosome sequences reveals the Japanese population history in the Jomon period. Scientific Reports, 9. ARTN 8556. 10.1038/s41598-019-44473-z PMC6572846 31209235

[ref101] Weissensteiner, H., Pacher, D., Kloss-Brandstätter, A., Forer, L., Specht, G., Bandelt, H.-J., … Schönherr, S. (2016). HaploGrep 2: Mitochondrial haplogroup classification in the era of high-throughput sequencing. Nucleic Acids Research, 44(W1), W58–W63. 10.1093/nar/gkw233 27084951 PMC4987869

[ref102] Whitman, J. (2011). Northeast Asian linguistic ecology and the advent of rice agriculture in Korea and Japan. Rice, 4(3), 149–158.

[ref103] Wollstein, A., & Lao, O. (2015). Detecting individual ancestry in the human genome. Investigative Genetics, 6, article #6. 10.1186/s13323-015-0019-x PMC4416275 25937887

[ref104] Wurm, S. A., & Hattori, S. (Eds.) (1981). Language Atlas of Pacific area, Canberra: Australian Academy of the Humanities.

[ref105] Yang, M. A., Gao, X., Theunert, C., Tong, H., Aximu Petri, A., Nickel, B., … Fu, Q. (2017). 40,000-year-old individual from Asia provides insight into early population structure in Eurasia. Current Biology, 27(20), 3202–3208 10.1016/j.cub.2017.09.030 29033327 PMC6592271

[ref106] Zhao, Y. B., Li, H. J., Cai, D. W., Li, C. X., Zhang, Q. C., Zhu, H., & Zhou, H. (2010). Ancient DNA from nomads in 2500-year-old archeological sites of Pengyang, China. Journal of Human Genetics, 55(4), 215–218. 20186156 10.1038/jhg.2010.8

