# Supplementary material for: Japan considered from the hypothesis of farmer/language spread
Source: Evol Hum Sci. 2020 May 5;2:e13. doi: 10.1017/ehs.2020.7 (PMC10427481; doi:10.1017/ehs.2020.7)
Supplement: Supplementary file 1 [file S2513843X20000079sup001.zip › Suppl figure captions & tables.docx]

**Japan considered from the hypothesis of farmer/language spread**

**de Boer, Elisabeth et al.,**

**SUPPLEMENTARY MATERIALS:**

**4 captions for supplementary figures (S1-S4); IMAGES BEING SENT SEPARATELY BY WETRANSFER**

**2 supplementary tables**

**Figure S1** Outgroup f_3_ test ranking the level of genetic similarity among different Asian populations (on the X-axis) to Hòabìnhians (G1) or the Ikawazu Jōmon, sorted from most genetic similarity (highest f_3_) to least genetic similarity (lowest f_3_, graph by MAY). The ordered values come from the test (A) *f_3_(Mbuti; ‘X’ Asians, Hòabìnhians)* or (B) *f_3_(Mbuti; ‘X’ Asians, Ikawazu Jōmon)*, where the Central African Mbuti was used as an outgroup population. Numerical values for the data visualized here can be found in Table S4.

KEY:

Two standard errors (SE) are indicated by the gray bar. The horizontal dashed gray line indicates the value for f_3_(Mbuti*, Ikawazu Jōmon,* *Hòabìnhians*), plus or minus two SE (0.254, SE=0.0035). Points are colored by region of origin, similar to the color coding in Figure 2, but with the addition of two additional groups, ancient Southeast Asian farmers (yellow) and ancient Asians with deep ancestry relative to East Asians (black). *Ancient islanders were sampled from Southeast Asian and Southwest Pacific islands, but were previously shown to share a close relationship to present-day Austronesians (McColl et al., 2018; Skoglund et al., 2016) and therefore also shares a close relationship to ancient East Asians emanating from the South China coast.

INTERPRETATION:

In Figure S1-A, the Hòabìnhians (G1) shares high genetic similarity with ancient Southeast Asian farmers, who were previously shown to share partial ancestry with Hòabìnhians (McColl et al., 2018). Notably, the Ikawazu Jōmon’s genetic similarity to Hòabìnhians is similar or lower to that of several present-day and ancient mainland East Asians.

In Figure S1-B, the Ikawazu Jōmon shares high genetic similarity with present-day Japanese, consistent with present-day Japanese possessing partial ancestry related to the Jōmon. The Hòabinhians’ (G1) genetic similarity to the Ikawazu Jōmon is very low, similar to other populations with ancestry deeply divergent from East Asians (black).

13MB, 2400 pixels across @ 600dpi = 4 inches

**Figure S2** Map of the Japanese languages and dialects
Original by Enirac Sum, translated by Zakuragi, CC A-S-A 3.0 unported https://creativecommons.org/licenses/by-sa/3.0/legalcode https://commons.wikimedia.org/w/index.php?curid=4425109

16.8MB, 2400 pixels across @ 300dpi = 8 inches

**Figure S3** Corner-projected mound-burials in Izumo and Koshi, square icons indicating sizes. Mound-burial illustration shows cobbled sides. Adapted by EdB & GLB from Torrance 2016, p. 15, and Wada 1998, fig. 12.

25.2MB, 3000 pixels across @ 600 dpi = 5 inches

**Figure S4** PCA comparing different populations in Japan. Tohoku cluster circled. Adapted by EdB from Saitō, 2017, p. 127.

17.8MB, 1800 pixels across @600 dpi = 3 inches

**Table S1**  **Features shared by Izumo, Noto and Tōhoku** (segmental phonology)

• Centralization of /i/, /u/, raising of /e/, /o/ (Black areas in Figure 5)
• Lowering of word initial /i/, and /i/ following vowels, resulting in a merger with /e/.

• Merger of /i/ and /u/ after coronal consonants (Striped areas in Figure 5)

**Table S2** **Features shared by Izumo and Tōhoku** (segmental and tonal phonology)

• Lowering of word-initial /u/, and /u/ in the first syllable after certain consonants resulting in a merger with /o/.
• Palatalization of /ki/ > [kɕi] or [tɕi]
• Merger of tone classes 2.1 and 2.2 and classes 3.1 and 3.2. (Green areas Figure 4)

• Shift of H tone away from /i/ and /u/. (Horizontal striped green areas Figure 4) Replacement of the imperative suffix -ro of vowel stem verbs with -re.

**Table S3 Data visualized in Fig. 2.** The shaded gray cells are ancient and present-day coastal populations off the coast of mainland Asia. Meaning and interpretation are as described in Fig. 2. All data came from previously published papers, compiled in Table S5. For the f_4_-analysis, we used Admixtools, version 412 and the *qpDstat* software with “f4mode: YES” (Patterson, et al., 2012).

| **X** | **A. f_4_(Mbuti, Hòabìnhian; Ikawazu, X)** | | **B. f_4_(Mbuti, Ikawazu; Chokhopani, X)** | | **C. f_4_(Mbuti, Ikawazu; Shamanka_EN, X)** | |
| --- | --- | --- | --- | --- | --- | --- |
|  | **f_4_** | **SE** | **f_4_** | **SE** | **f_4_** | **SE** |
| **Chokhopani** | 0.0006 | 0.0006 | - | - | -0.0002 | 0.0005 |
| **Mebrak** | 0.0002 | 0.0006 | 0.0003 | 0.0006 | 0.0003 | 0.0005 |
| **Samdzong** | -0.0006 | 0.0005 | -0.0001 | 0.0005 | -0.0003 | 0.0004 |
| **Tibetan** | -0.0006 | 0.0005 | -0.0001 | 0.0005 | -0.0004 | 0.0004 |
| **Sherpa** | -0.0008 | 0.0005 | -0.0004 | 0.0005 | -0.0006 | 0.0004 |
| **Shamanka_EN** | -0.0001 | 0.0008 | 0.0002 | 0.0005 | - | - |
| **Lokomotiv_EN** | -0.0003 | 0.0005 | 0.0008 | 0.0005 | 0.0006 | 0.0003 |
| **UstBelaya_N** | -0.0007 | 0.0005 | 0.0014 | 0.0007 | 0.0012 | 0.0005 |
| **DevilsCave_N** | -0.0005 | 0.0005 | 0.0022 | 0.0005 | 0.002 | 0.0003 |
| **Xibo** | -0.0011 | 0.0005 | 0.0008 | 0.0005 | 0.0005 | 0.0004 |
| **Hezhen** | -0.0002 | 0.0005 | 0.0011 | 0.0005 | 0.0008 | 0.0003 |
| **Daur** | -0.0012 | 0.0006 | 0.0007 | 0.0006 | 0.0004 | 0.0004 |
| **Japanese** | 0.0001 | 0.0005 | 0.0054 | 0.0005 | 0.0052 | 0.0003 |
| **Korean** | 0.0001 | 0.0005 | 0.0018 | 0.0005 | 0.0016 | 0.0003 |
| **Han** | -0.0002 | 0.0005 | 0.0014 | 0.0005 | 0.0012 | 0.0003 |
| **Vanuatu** | -0.0002 | 0.0008 | 0.0007 | 0.0007 | 0.0001 | 0.0005 |
| **G6** | -0.0003 | 0.0007 | 0.0005 | 0.0006 | 0.0003 | 0.0005 |
| **Ami** | 0.0006 | 0.0005 | 0.0026 | 0.0005 | 0.0023 | 0.0004 |
| **Atayal** | -0.0003 | 0.0006 | 0.0021 | 0.0006 | 0.0019 | 0.0005 |
| **Lahu** | -0.0002 | 0.0005 | 0.0007 | 0.0005 | 0.0005 | 0.0004 |
| **Dai** | 0.0001 | 0.0005 | 0.001 | 0.0005 | 0.0008 | 0.0003 |
| **Kinh** | 0 | 0.0005 | 0.0006 | 0.0005 | 0.0004 | 0.0004 |

**Table S4 Data visualized in Fig. S1. Meaning and interpretation are as described in Fig. S1.** All data came from previously published papers, compiled in Table S5. For the f_3_-analysis, we used Admixtools and the *qp3pop* software with default parameters (version 412, Patterson, et al., 2012).

| **f_3_(Mbuti; Hòabìnhian – G1, X)** | | | | 1. **f_3_(Mbuti; Ikawazu, X)** | | | |
| --- | --- | --- | --- | --- | --- | --- | --- |
| **X** | **f3** | **SE** | **N** | **X** | **f3** | **SE** | **N** |
| **Vt_G2** | 0.2648 | 0.0044 | 73244 | **Japanese** | 0.3003 | 0.0034 | 679622 |
| **G5** | 0.2628 | 0.0054 | 46008 | **Ulchi** | 0.2898 | 0.0033 | 664078 |
| **Ban_Chiang** | 0.2628 | 0.0061 | 28921 | **Ami** | 0.2884 | 0.0034 | 661730 |
| **G3** | 0.2617 | 0.0035 | 156958 | **DevilsCave_N** | 0.2870 | 0.0034 | 650953 |
| **Nui_Nap** | 0.2592 | 0.0040 | 107253 | **Atayal** | 0.2866 | 0.0035 | 635961 |
| **Oakaie1** | 0.2585 | 0.0047 | 63426 | **Korean** | 0.2852 | 0.0034 | 665630 |
| **G4** | 0.2580 | 0.0034 | 208661 | **She** | 0.2844 | 0.0033 | 663976 |
| **G6** | 0.2580 | 0.0036 | 182423 | **Tujia** | 0.2841 | 0.0034 | 665295 |
| **Ma912_G2** | 0.2580 | 0.0034 | 286653 | **UstBelaya_N** | 0.2836 | 0.0038 | 262889 |
| **UstBelaya_N** | 0.2575 | 0.0038 | 152176 | **Han** | 0.2835 | 0.0033 | 697910 |
| **La_G2** | 0.2573 | 0.0034 | 297744 | **Oroqen** | 0.2828 | 0.0033 | 665190 |
| **Ami** | 0.2558 | 0.0031 | 380309 | **G6** | 0.2824 | 0.0036 | 311219 |
| **Yi** | 0.2553 | 0.0031 | 383139 | **Hezhen** | 0.2821 | 0.0033 | 665000 |
| **Man_Bac** | 0.2551 | 0.0038 | 101955 | **Naxi** | 0.2820 | 0.0033 | 682627 |
| **Mebrak** | 0.2546 | 0.0035 | 228240 | **Dai** | 0.2819 | 0.0033 | 699334 |
| **Chokhopani** | 0.2545 | 0.0033 | 353824 | **Miao** | 0.2817 | 0.0034 | 664671 |
| **She** | 0.2545 | 0.0031 | 381929 | **Lokomotiv_EN** | 0.2813 | 0.0034 | 559798 |
| **Japanese** | 0.2544 | 0.0031 | 391602 | **Yi** | 0.2812 | 0.0033 | 666511 |
| **Korean** | 0.2544 | 0.0031 | 382789 | **Xibo** | 0.2808 | 0.0034 | 667039 |
| **Dai** | 0.2541 | 0.0031 | 402021 | **Oakaie1** | 0.2808 | 0.0041 | 106418 |
| **Kinh** | 0.2539 | 0.0031 | 382630 | **Lahu** | 0.2806 | 0.0034 | 663695 |
| **Ikawazu** | 0.2538 | 0.0035 | 280931 | **Daur** | 0.2804 | 0.0036 | 639242 |
| **Tujia** | 0.2536 | 0.0032 | 382445 | **Kinh** | 0.2801 | 0.0034 | 665782 |
| **Naxi** | 0.2536 | 0.0031 | 392391 | **Mebrak** | 0.2795 | 0.0036 | 398544 |
| **Han** | 0.2531 | 0.0031 | 401220 | **La_G2** | 0.2790 | 0.0035 | 512351 |
| **Lahu** | 0.2530 | 0.0031 | 381328 | **Nui_Nap** | 0.2788 | 0.0039 | 187856 |
| **Vanuatu** | 0.2530 | 0.0039 | 110660 | **Shamanka_EN** | 0.2786 | 0.0032 | 691106 |
| **Ulchi** | 0.2530 | 0.0032 | 382377 | **Chokhopani** | 0.2776 | 0.0035 | 617018 |
| **Hezhen** | 0.2529 | 0.0032 | 382501 | **Samdzong** | 0.2773 | 0.0033 | 636813 |
| **DevilsCave_N** | 0.2529 | 0.0032 | 374320 | **Vt_G2** | 0.2772 | 0.0042 | 115581 |
| **Miao** | 0.2524 | 0.0031 | 382152 | **Tibetan** | 0.2770 | 0.0034 | 666347 |
| **Oroqen** | 0.2521 | 0.0031 | 382596 | **Vanuatu** | 0.2768 | 0.0036 | 203005 |
| **Atayal** | 0.2521 | 0.0033 | 365416 | **G3** | 0.2765 | 0.0035 | 249963 |
| **Sherpa** | 0.2515 | 0.0031 | 381565 | **Sherpa** | 0.2761 | 0.0034 | 663936 |
| **Lokomotiv_EN** | 0.2514 | 0.0032 | 323764 | **G4** | 0.2752 | 0.0033 | 353600 |
| **Shamanka_EN** | 0.2513 | 0.0030 | 397541 | **Ma912_G2** | 0.2748 | 0.0036 | 491702 |
| **Samdzong** | 0.2511 | 0.0032 | 365512 | **Kolyma** | 0.2740 | 0.0037 | 617786 |
| **Tibetan** | 0.2508 | 0.0031 | 382813 | **Man_Bac** | 0.2730 | 0.0037 | 177923 |
| **Xibo** | 0.2490 | 0.0031 | 383427 | **G5** | 0.2676 | 0.0046 | 78520 |
| **Daur** | 0.2480 | 0.0032 | 367328 | **USR1** | 0.2671 | 0.0036 | 618212 |
| **USR1** | 0.2478 | 0.0033 | 354533 | **Ban_Chiang** | 0.2612 | 0.0049 | 48160 |
| **Kolyma** | 0.2469 | 0.0034 | 354384 | **G1** | 0.2538 | 0.0035 | 280931 |
| **Tianyuan** | 0.2447 | 0.0034 | 261212 | **Tianyuan** | 0.2485 | 0.0035 | 473358 |

**Table S5 Names of ancient individuals and present-day populations included in Fig. 2 and S1.** All are previously published, with references noted in the final column. Data in a format that can be read by the software Admixtools (version 412, Patterson, et al., 2012) where genotyped data are available for the ‘1240K’ SNP panel can be found at the link below the table*. Data for Sikora, et al., (2019) were reformatted to follow the same data structure and included with the larger dataset.

| **Name** | **Region** | **# Samples** | **# SNPs** | **References** |
| --- | --- | --- | --- | --- |
| Tianyuan | East Asia (Beijing) | 1 | 958361 | Yang, et al., 2017 |
| Hòabìnhian (G1^) | Southeast Asia | 2 | 534536 | McColl, et al., 2018 |
| Ikawazu | Japanese Archipelago (island) | 1 | 928906 | McColl, et al., 2018 |
| Kolyma | northern Siberia | 1 | 1231156 | Sikora, et al., 2019 |
| Shamanka_EN | Siberia | 10 | 1230495 | de Barros Damgaard, et al., 2018 |
| Lokomotiv_EN | Siberia | 4 | 1063593 | de Barros Damgaard, et al., 2018 |
| UstBelaya_N | Siberia | 1 | 508972 | Sikora, et al., 2019 |
| DevilsCave_N | Siberia | 6 | 1201365 | Sikora, et al., 2019 |
| Chokhopani | Tibetan Plateau | 1 | 1226443 | Jeong, et al., 2016 |
| Mebrak | Tibetan Plateau | 1 | 768444 | Jeong, et al., 2016 |
| Samdzong | Tibetan Plateau | 2 | 1225761 | Jeong, et al., 2016 |
| Vanuatu | Southwest Pacific island | 3 | 377379 | Skoglund, et al., 2016 |
| G6^ | Southeast Asian island | 3 | 599658 | McColl, et al., 2018 |
| Man_Bac | Southeast Asia | 8 | 330539 | Lipson, et al., 2018 |
| Nui_Nap | Southeast Asia | 2 | 353311 | Lipson, et al., 2018 |
| Ban_Chiang | Southeast Asia | 5 | 89398 | Lipson, et al., 2018 |
| Oakaie1 | Southeast Asia | 2 | 199739 | Lipson, et al., 2018 |
| G3^ | Southeast Asia | 5 | 476875 | McColl, et al., 2018 |
| G4^ | Southeast Asia | 4 | 675968 | McColl, et al., 2018 |
| Ma912_G2^ | Southeast Asia | 1 | 955193 | McColl, et al., 2018 |
| La_G2^ | Southeast Asia | 3 | 986469 | McColl, et al., 2018 |
| Vt_G2^ | Southeast Asia | 2 | 219388 | McColl, et al., 2018 |
| Simons Genome Diversity Panel: present-day East Asians (Mallick, et al., 2016) | | | | |
| Ulchi | Japanese | Ami | Naxi | Tibetan |
| Oroqen | Korean | Atayal | Yi | Sherpa |
| Xibo | Han | Lahu | Tujia |  |
| Daur | Miao | Kinh |  |  |
| Hezhen | She | Dai |  |  |
| *<https://reich.hms.harvard.edu/downloadable-genotypes-present-day-and-ancient-dna-data-compiled-published-papers>  ^Individuals from McColl, et al., (2018) were grouped by genetic patterns and given names of Group 1 (G1), Group 2 (G2), etc. The nomenclature above groups those individuals into one set for analysis to increase the number of SNPs available for comparison. La and Vt indicate individuals sampled in Laos or individuals sampled in Vietnam, respectively. | | | | |
