# Supplementary figures and images for: Japan considered from the hypothesis of farmer/language spread
Source: Evol Hum Sci. 2020 May 5;2:e13. doi: 10.1017/ehs.2020.7 (PMC10427481; doi:10.1017/ehs.2020.7)

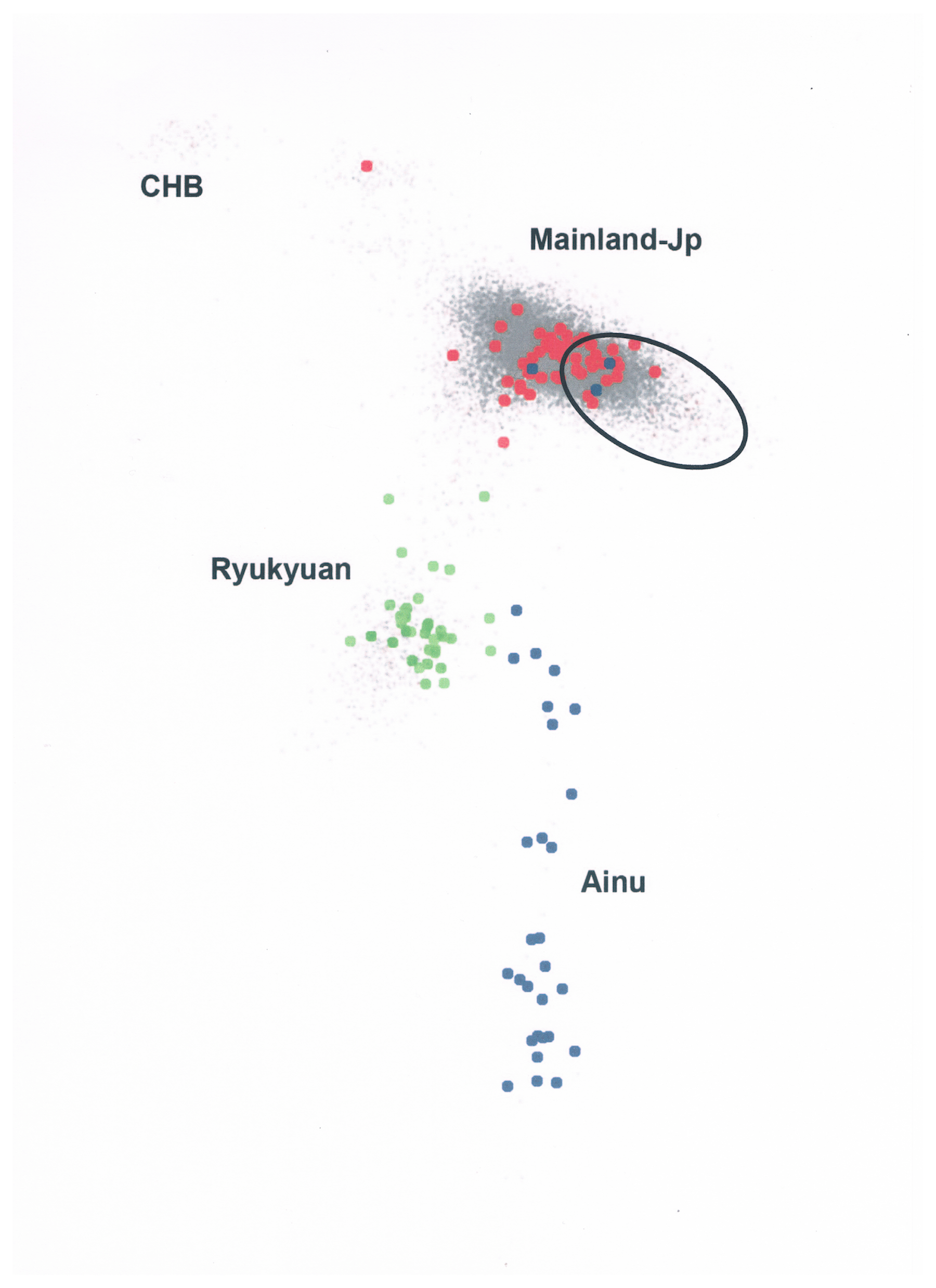

Supplement: Supplementary file 1 [file S2513843X20000079sup001.zip › deBoer Fig.S4 TohokuDNA_color.tiff]

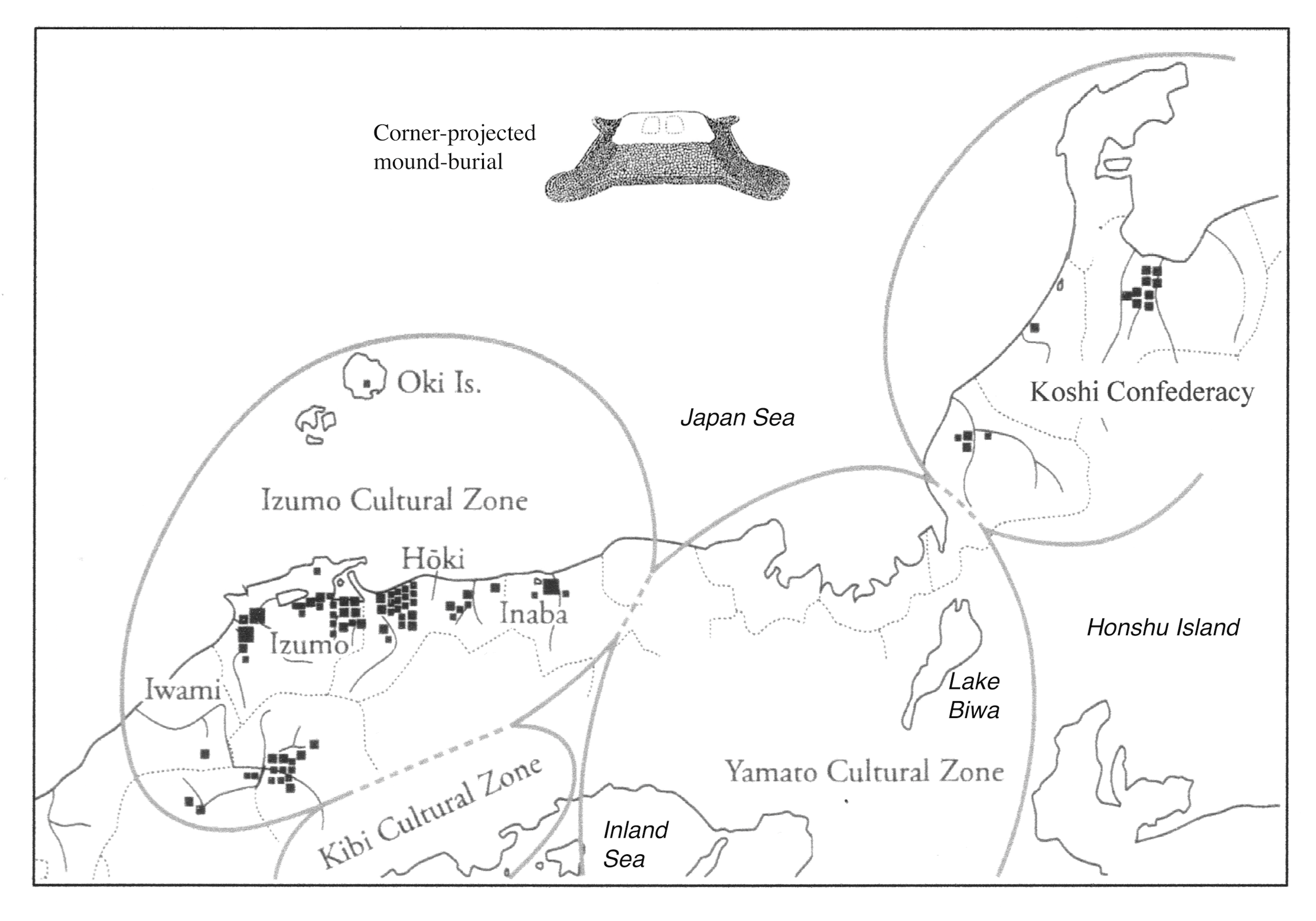

Supplement: Supplementary file 1 [file S2513843X20000079sup001.zip › deBoer Fig.S3_Koshi Confederacy w_pic&.tiff]

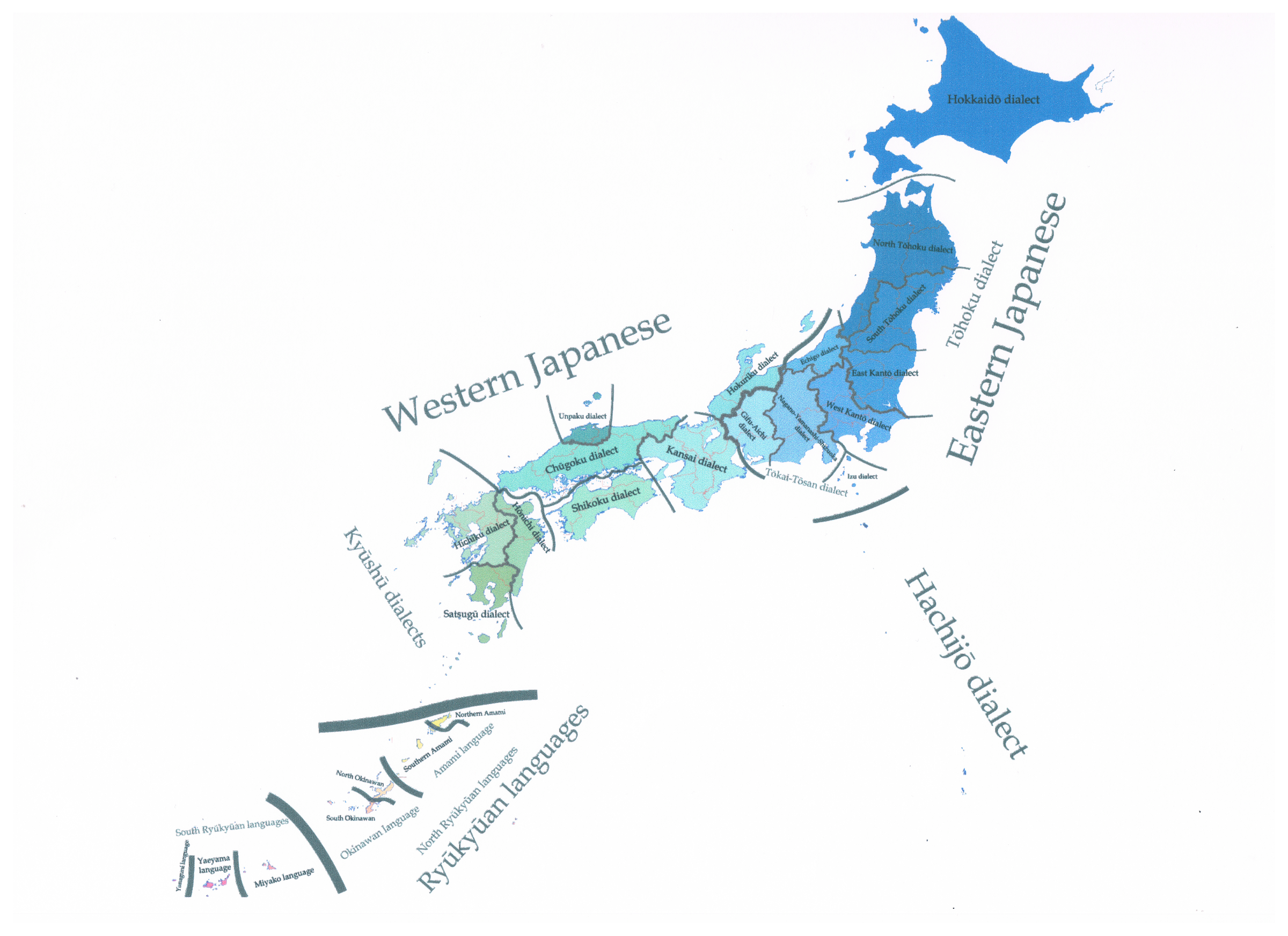

Supplement: Supplementary file 1 [file S2513843X20000079sup001.zip › deBoer Fig.S2 Japanese dialect map_color.tiff]

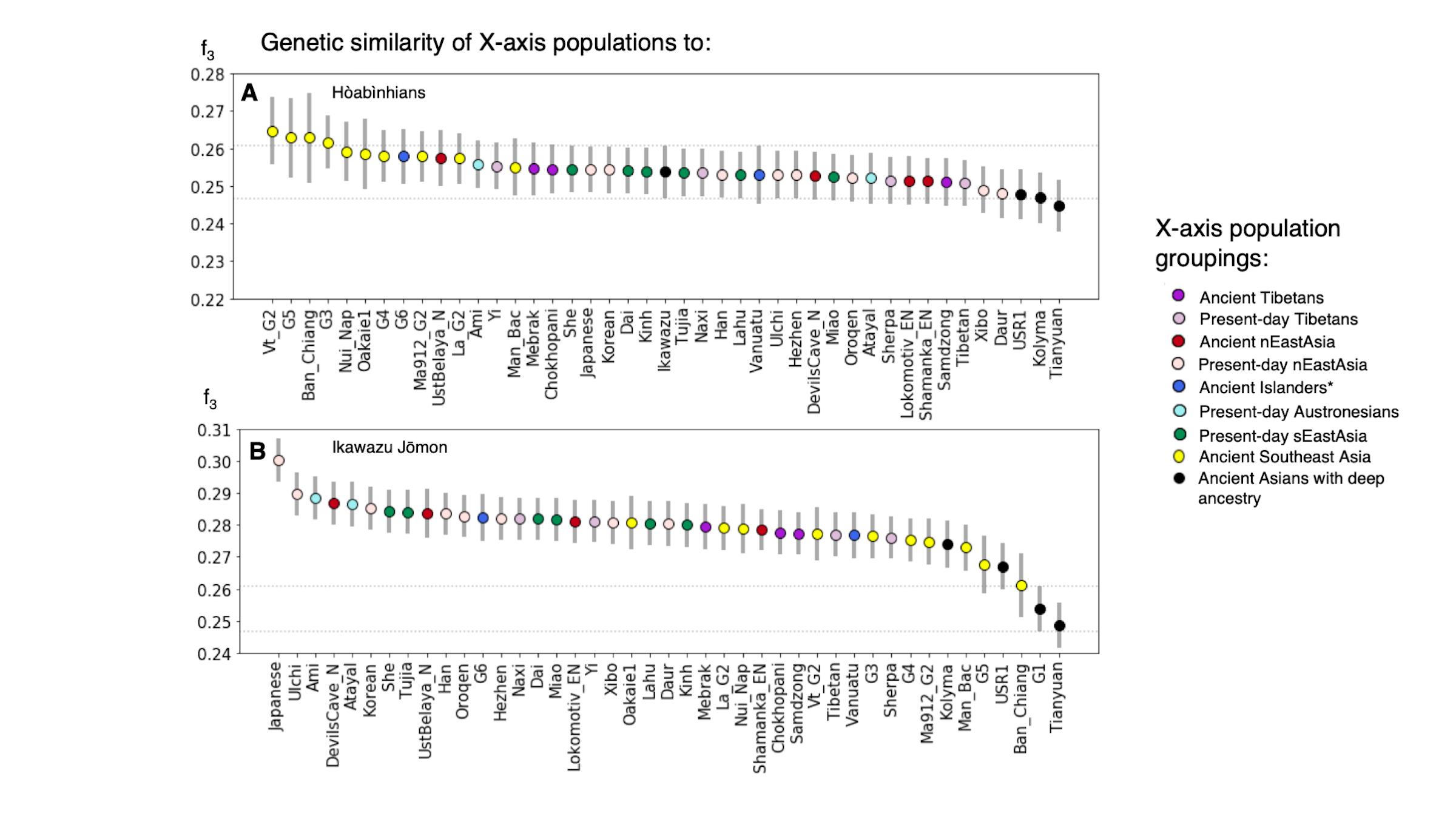

Supplement: Supplementary file 1 [file S2513843X20000079sup001.zip › deBoer Fig.S1 2-way f3 genetic tests.tiff]
